# Supplementary material for: Monitoring physiological processes of fast-growing broilers during the whole life cycle: Changes of redox-homeostasis effected to trassulfuration pathway predicting the development of non-alcoholic fatty liver disease
Source: PLoS One. 2023 Aug 17;18(8):e0290310. doi: 10.1371/journal.pone.0290310 (PMC10434899; doi:10.1371/journal.pone.0290310)
Supplement: Attachment — Submitted filename: PONE-D-23-15244.pdf [file pone.0290310.s001.pdf]

PLOS ONE

Monitoring physiological processes of fast-growing broilers during the whole life cycle: changes of redox-homeostasis effected to trassulfuration pathway predicting the development of non-alcoholic fatty liver disease

--Manuscript Draft--

Manuscript Number:	PONE-D-23-15244
Article Type:	Research Article
Full Title:	Monitoring physiological processes of fast-growing broilers during the whole life cycle: changes of redox-homeostasis effected to trassulfuration pathway predicting the development of non-alcoholic fatty liver disease
Short Title:	Diagnostic relevance of the redox status in fast-growing broilers during rearing
Corresponding Author:	Judit Remenyik, PhD<br>University of Debrecen: Debreceni Egyetem<br>Debrecen, HUNGARY
Keywords:	broiler, antioxidants, transsulfuration pathway, non-alcoholic fatty liver disease
Abstract:	In the broiler industry, the average daily gain and feed conversion ratio are extremely favorable, but the birds are beginning to approach the maximum of their genetic capacity. However, as a consequence of strong genetic selection, the occurrence of certain metabolic diseases, such as myopathies, ascites, sudden cardiac death and tibial dyschondroplasia, is increasing.<br>These metabolic diseases can greatly affect the health status and welfare of birds, as well as the quality of meat.<br>The main goal of this study was to investigate the changes in the main parameters of redox homeostasis during the rearing (1-42 days of age) of broilers with high genetic capacity, such as the concentrations of malondialdehyde, vitamin C, vitamin E, and reduced glutathione, the activities of glutathione peroxidase and glutathione reductase, and the inhibition rate of superoxide dismutase. Damage to the transsulfuration pathway during growth and the reason for changes in the level of homocysteine were investigated. Further, the parameters that can characterize the biochemical changes occurring in the birds were examined. Our study is the first characterize plasma albumin saturation. A method was developed to measure the levels of other small molecule thiol components of plasma. Changes in redox homeostasis induce increases in the concentrations of tumor necrosis factor alpha and inflammatory interleukins interleukin 2, interleukin 6 and interleukin 8 in broilers reared according to current large-scale husbandry technology and feeding protocols. A significant difference in all parameters tested was observed on the 21st day. The concentrations of cytokines and homocysteine increased, while the concentrations of glutathione and cysteine in the plasma decreased.<br>Our findings suggest that observed changes in the abovementioned biochemical indices have a negative effect on poultry health.
Order of Authors:	Judit Remenyik, PhD
	Georgina Pesti-Asbóth
	Endre Szilágyi
	Piroska Bíróné Molnár
	János Oláh, PhD
	László Babinszky, PhD
	Levente Czeglédi, PhD
	Cziáky Zoltán, PhD
	Melinda Paholcsek, PhD
	László Stündl, PhD
Additional Information:	

Powered by Editorial Manager® and ProduXion Manager® from Aries Systems Corporation

Question	Response
**Financial Disclosure**<br><br>Enter a financial disclosure statement that describes the sources of funding for the work included in this submission. Review the submission guidelines for detailed requirements. View published research articles from *PLOS ONE* for specific examples.<br><br>This statement is required for submission and **will appear in the published article** if the submission is accepted. Please make sure it is accurate.<br><br>**Unfunded studies**<br>Enter: *The author(s) received no specific funding for this work.*<br><br>**Funded studies**<br>Enter a statement with the following details:<br>• Initials of the authors who received each award<br>• Grant numbers awarded to each author<br>• The full name of each funder<br>• URL of each funder website<br>• Did the sponsors or funders play any role in the study design, data collection and analysis, decision to publish, or preparation of the manuscript?<br>• **NO** - Include this sentence at the end of your statement: *The funders had no role in study design, data collection and analysis, decision to publish, or preparation of the manuscript.*<br>• **YES** - Specify the role(s) played.<br><br>* typeset	This study was financially supported by the Gazdaságfejlesztési és Innovációs Operatív Program (GINOP) GINOP-2.3.2-15-2016-00042 project of the Széchenyi 2020 Program given by the European Union and the Hungarian Government
**Competing Interests**<br><br>Use the instructions below to enter a competing interest statement for this submission. On behalf of all authors, disclose any competing interests that could be perceived to bias this work—acknowledging all financial support and any other relevant financial or non-financial competing interests.	The authors have declared that no competing interests exist.

This statement is required for submission and **will appear in the published article** if the submission is accepted. Please make sure it is accurate and that any funding sources listed in your Funding Information later in the submission form are also declared in your Financial Disclosure statement.<br><br>View published research articles from *PLOS ONE* for specific examples.<br><br>**NO authors have competing interests**<br><br>Enter: *The authors have declared that no competing interests exist.*<br><br>**Authors with competing interests**<br><br>Enter competing interest details beginning with this statement:<br><br>*I have read the journal's policy and the authors of this manuscript have the following competing interests: [insert competing interests here]*<br><br>* typeset	
**Ethics Statement**<br><br>Enter an ethics statement for this submission. This statement is required if the study involved:<br><br>• Human participants<br>• Human specimens or tissue<br>• Vertebrate animals or cephalopods<br>• Vertebrate embryos or tissues<br>• Field research<br><br>Write "N/A" if the submission does not require an ethics statement.<br><br>General guidance is provided below. Consult the submission guidelines for detailed instructions. **Make sure that all information entered here is included in the Methods section of the manuscript.**	The study was conducted with the approval of the local ethics committee of the University of Debrecen (DEMAB/12-7/2015).

Powered by Editorial Manager® and ProduXion Manager® from Aries Systems Corporation

**Format for specific study types**<br><br>**Human Subject Research (involving human participants and/or tissue)**<br>• Give the name of the institutional review board or ethics committee that approved the study<br>• Include the approval number and/or a statement indicating approval of this research<br>• Indicate the form of consent obtained (written/oral) or the reason that consent was not obtained (e.g. the data were analyzed anonymously)<br><br>**Animal Research (involving vertebrate animals, embryos or tissues)**<br>• Provide the name of the Institutional Animal Care and Use Committee (IACUC) or other relevant ethics board that reviewed the study protocol, and indicate whether they approved this research or granted a formal waiver of ethical approval<br>• Include an approval number if one was obtained<br>• If the study involved *non-human primates*, add *additional details* about animal welfare and steps taken to ameliorate suffering<br>• If anesthesia, euthanasia, or any kind of animal sacrifice is part of the study, include briefly which substances and/or methods were applied<br><br>**Field Research**<br><br>Include the following details if this study involves the collection of plant, animal, or other materials from a natural setting:<br>• Field permit number<br>• Name of the institution or relevant body that granted permission	
**Data Availability**<br><br>Authors are required to make all data underlying the findings described fully available, without restriction, and from the time of publication. PLOS allows rare exceptions to address legal and ethical concerns. See the PLOS Data Policy and FAQ for detailed information.	Yes - all data are fully available without restriction

A Data Availability Statement describing where the data can be found is required at submission. Your answers to this question constitute the Data Availability Statement and **will be published in the article**, if accepted.<br><br>**Important:** Stating 'data available on request from the author' is not sufficient. If your data are only available upon request, select 'No' for the first question and explain your exceptional situation in the text box.<br><br>Do the authors confirm that all data underlying the findings described in their manuscript are fully available without restriction?	
**Describe where the data may be found in full sentences. If you are copying our sample text, replace any instances of XXX with the appropriate details.**<br><br>• If the data are **held or will be held in a public repository**, include URLs, accession numbers or DOIs. If this information will only be available after acceptance, indicate this by ticking the box below. For example: *All XXX files are available from the XXX database (accession number(s) XXX, XXX.*).<br>• If the data are all contained **within the manuscript and/or Supporting Information files**, enter the following: *All relevant data are within the manuscript and its Supporting Information files.*<br>• If neither of these applies but you are able to provide **details of access elsewhere**, with or without limitations, please do so. For example:<br><br>*Data cannot be shared publicly because of [XXX]. Data are available from the XXX Institutional Data Access / Ethics Committee (contact via XXX) for researchers who meet the criteria for access to confidential data.*<br><br>*The data underlying the results presented in the study are available*	All relevant data are within the manuscript and its Supporting Information files.

from (include the name of the third party and contact information or URL).<br>• This text is appropriate if the data are owned by a third party and authors do not have permission to share the data.<br><br>* typeset	
Additional data availability information:	Tick here if your circumstances are not covered by the questions above and you need the journal’s help to make your data available.

Click here to access/download;Manuscript;Manuscript.docx

Monitoring physiological processes of fast-growing broilers during the whole life cycle: changes of redox-homeostasis effected to trassulfuration pathway predicting the development of non-alcoholic fatty liver disease

Diagnostic relevance of the redox status in fast-growing broilers during rearing

Georgina Pesti-Asbóth[1,] EndreSzilágyi[1], Piroska BírónéMolnár[1],János Oláh[2], László Babinszky[3], Levente Czeglédi[4], Zoltán Cziáky[5], Melinda Paholcsek[1], László Stündl[1]and Judit Remenyik[1]

[1] Institute of Food Technology, Faculty of Agricultural and Food Sciences and Environmental Management, University of Debrecen, Debrecen, Hungary

[2] Farm and Regional Research Institute of Debrecen, University of Debrecen, Debrecen, Hungary

[3] Department of Animal Nutrition Physiology, Institute of Animal Science, Biotechnology and Nature Conservation, Faculty of Agricultural and Food Sciences and Environmental Management, University of Debrecen, Debrecen, Hungary

[4] Department of Animal Science, Institute of Animal Science, Biotechnology and Nature Conservation, Faculty of Agricultural and Food Sciences and Environmental Management, University of Debrecen, Debrecen, Hungary

[5] Agricultural and Molecular Research and Service Group, University of Nyíregyháza; Nyíregyháza, Hungary

* remenyik@agr.unideb.hu (J.R.)

Abstract

In the broiler industry, the average daily gain and feed conversion ratio are extremely favorable, but the birds are beginning to approach the maximum of their genetic capacity. However, as a consequence of strong genetic selection, the occurrence of certain metabolic diseases, such as myopathies, ascites, sudden cardiac death and tibial dyschondroplasia, is increasing.

These metabolic diseases can greatly affect the health status and welfare of birds, as well as the quality of meat.

The main goal of this study was to investigate the changes in the main parameters of redox homeostasis during the rearing (1-42 days of age) of broilers with high genetic capacity, such as the concentrations of malondialdehyde, vitamin C, vitamin E, and reduced glutathione, the activities of glutathione peroxidase and glutathione reductase, and the inhibition rate of superoxide dismutase. Damage to the transsulfuration pathway during growth and the reason for changes in the level of homocysteine were investigated. Further, the parameters that can characterize the biochemical changes occurring in the birds were examined. Our study is the first characterize plasma albumin saturation. A method was developed to measure the levels of other small molecule thiol components of plasma. Changes in redox homeostasis induce increases in the concentrations of tumor necrosis factor alpha and inflammatory interleukins interleukin 2, interleukin 6 and interleukin 8 in broilers reared according to current large-scale husbandry technology and feeding protocols. A significant difference in all parameters tested was observed on the 21st day. The concentrations of cytokines and homocysteine increased, while the concentrations of glutathione and cysteine in the plasma decreased.

Our findings suggest that observed changes in the abovementioned biochemical indices have a negative effect on poultry health.

Introduction

The main issues that burden the broiler industry today might be caused by genetic selection strategies, such as a rapid growth rate, a decreasing slaughter age and facilitating feed efficiency [1]. Indeed, achieving extreme growth potential is a serious biotic stress factor that is accompanied by health problems leading to systemic diseases [2,3].

The path mechanisms of most common infectious diseases, including pneumonia, sepsis and enteritis, are directly linked to the failure of redox homeostasis [4–7]. According to the latest research findings, a high susceptibility to pathogens was closely related to oxidative stress in 308 Ross broiler chickens [8,9]. In addition, fatal complications, such as acidosis, sudden cardiac death, dyschondroplasia or wooden breast (WB) syndrome, have been proven to be a result of genetics along with additional unknown causes[10,11]. Therefore, revealing potential tools to avoid the accumulation of genetic alterations has recently become crucial.

In addition to being an important economic factor, the present condition of the poultry industry has drawn attention to global issues that must be solved, such as animal welfare. For instance, limiting the use of antibiotics seems to be challenging because genetically modified poultry strains are highly susceptible to infections[12].

Pathological alterations in the cardiovascular system and the liver are as important as infections in terms of the sustainability of the poultry industry. WB myopathy is a systemic disease in which complex metabolic and physiological changes have been implicated that is characterized by hardened areas, white striping and small hemorrhages in the pectoralis major muscle[13]. Macroscopic changes might develop due to the abnormal accumulation of endomysial and perimysial connective tissue, which in turn can lead to fibrosis, hypoxia, oxidative stress and the activation of inflammatory pathways[13–16]. Xing et al. [17]noted that WB syndrome is strongly correlated with hepatocyte damage and elevated serum levels of

aspartate aminotransferase (AST) and γ-glutamyl transferase (GGT) accompanied by a high systemic inflammatory cytokine load. In addition, a high concentration of homocysteine (Hcy) was detected in animals suffering from myopathy compared to healthy chickens [18].

Other multifactorial diseases that affect production traits are ascites (AS) and sudden death syndrome, which are highly correlated with each other [19]. Cardiac arrest could also occur owing to hyperlipidemia-mediated atherosclerosis [20,21]. These conditions are often accompanied by liver fibrosis, which was originally common among birds bred in high-altitude areas. Currently, liver fibrosis has also become widespread in industrial farming, probably because of a deficiency in oxygen consumption that has been linked to the fast growth rate[22]. Severe hepatic impairment was reported in broilers with cold-induced ascites [23]. In addition, chronic inflammatory events have resulted in metabolic imbalance, as indicated by observed threefold elevation in serum Hcy [24].

During broiler growth, it is important to monitor how the antioxidant system can be characterized during each development cycle and how it correlates with the events in the transsulfuration pathway. To draw a more complete picture, it may be important to examine new parameters influencing the redox system. Recent research in the human body has shown that the small molecule thiol compounds in albumin and plasma significantly, if not mostly, determine plasma redox homeostasis [25,26]. Although it may seem that this is not of great importance from an economic point of view, it is possible to prevent the phenotypic appearance of certain diseases by monitoring changes in biochemical parameters.

Worldwide, remediation of the untenable circumstances in the broiler industry has already started in the fields of health care management, environmental inspections and animal welfare accreditation systems, but the key points in bird growth where the prevention or reversal of malformations is feasible are still not clear.[19,27,28] Proper livestock farming technology is essential for minimizing abiotic stress factors, but limiting biotic stress is also important[29],

and the latter is unattainable without revealing the mechanisms behind the development of genetic diseases.

In fact, novel biomarkers need to be found to facilitate the development of the poultry sector. This might also pave the way for the application of herbal substances as effective compounds that can modulate abovementioned molecular targets and in turn promote antibiotic-free livestock care when considering animal welfare and health protection. Therefore, the aim of the present study was to determine how the antioxidant status of fast-growing broilers changes during their lifecycle and what kind of enzymes (glutathione peroxidase and glutathione reductase) are activated to maintain homeostasis.

A further aim was to study how the transsulfuration pathway (TSP) is connected with the antioxidant protection system and its relevance in diagnostics.

Materials and Methods

Ethical Approval

The study was conducted with the approval of the local ethics committee of the University of Debrecen (DEMAB/12-7/2015).

Birds, bird housing, experimental design, and diet

A total of 180 1-day-old fast-growing broilers of both sexes from a commercial hatchery in Hungary were used in this study. The experiments were carried out on the experimental farm of the University of Debrecen. All broilers were placed in the same barn. Chickens were kept in floor pens covered with wood shavings in a thermostatically controlled house at a stocking density of 650 cm^2/bird. The temperature was 32 °C at placement and gradually decreased by 1.5 °C/week. The birds were exposed to light according to Olanrewaju et al. (2006) as follows: 23 L:1D during the first 7 days, 20 L:4D between Days 8−28 and 23 L:1D between Days 29−42 (L = light, D = dark)[30].

The 1-day-old chickens were randomly placed into 3 pens (60 birds/pen). The experiment started at 1 day of age and lasted until 42 days of age. Broilers were fed a commercial maize–soybean-based basal diet (BD) free of antibiotics according to four feeding periods: prestarter

(1 to 9 days), starter (10 to 21 days), grower (22 to 31 days), and finisher (32 to 42 days). All diets were fed in mash form. The composition and energy and nutrient contents of the diets are given in Table 1.

Diets and water were available *ad libitum* during the entire experiment. Broilers were weighed at 1, 10, 21, 32, and 42 days of age at which times the growth performance parameters, average body weight (BW), average daily gain (ADG), average daily feed intake (ADFI) and feed conversion ratio (FCR) were determined. The BW and ADG were measured individually, while the ADFI and FCR were calculated by pen at 1, 10, 21, 32 and 42 days of age.

The animals were sacrificed by cervical dislocation. Blood and liver samples were collected from 1 bird per pen except for on Day 3, when 3 birds/pen were processed. Blood samples from Day 3 were pooled for the experiments.

Table 1. Ingredients and chemical compositions of the basal diets fed during the prestarting (1-9 days), starting (10-21 days), growing (22-35 days) and finishing (35-42 days) phases

**Ingredient (%)**	**Prestarter (Days 1-9)**	**Starter (Days 10-21)**	**Grower (Days 10-21)**	**Finisher (Days 32-42)**
Corn	33	34	33	32
Wheat	27	29	31	32
Soybean meal, solvent extracted (46.0% CP[a])	29	24	20	16
Soybean meal, extruded (46.0%CP)	4	6	4	4
Sunflower meal, extracted	-	1	3	4
Feed yeast	1	-	-	-
Distillers-dried grains with solubles (DDGS)	-	1	3	5

Plant fats	2	1	3	4
Premix	4	4	3	3
Total	100	100	100	100
**Energy and nutrients in the diet**				
Dry matter, %	89.06	89.03	89.15	89.15
AME_n [b], Poultry, MJ/kg	12.23	12.47	12.81	13.01
Crude protein, %	21.58	20.28	19.05	18.28
Crude fat, %	4.61	4.83	6.22	6.83
Crude fiber, %	3.37	3.51	3.7	3.88
Lysine, %	1.37	1.27	1.17	1.09
Methionine, %	0.57	0.54	0.53	0.49
Methionine + cysteine, %	0.94	0.9	0.87	0.83
Calcium, %	0.85	0.73	0.71	0.67
Phosphorus, %	0.63	0.55	0.52	0.49
Sodium, %	0.17	0.16	0.16	0.16
Sodiumchloride, %	0.282	0.252	0.242	0.244
Vitamin A, mg/kg	12.500	12.500	12.500	8.750
Vitamin E, mg/kg	50.001	50.001	50.001	35
Vitamin D_3, mg/kg	3.000	3.000	3.000	2.100

CP, crude protein; AME_n, apparent metabolizable energy corrected for zero nitrogen balance.

The blood samples from Day 3 were pooled for the experiments. Blood was collected into EDTA-coated vacutainer tubes (BD, Franklin Lakes, NJ.). One milliliter of each sample was centrifuged at 1000 × g and4 °C for 10 min. The supernatant plasma was further divided into 300 µL aliquots and stored at -80 °C until analysis. Samples were used to determine the antioxidant status of the plasma.

Further, liver tissues were preserved in formalin (10% reagent grade for histology, neutral-buffered; VWR Hungary).

Determination of antioxidant parameters

Lipid peroxidation

Lipid peroxidation was determined using a commercially available assay kit (ab118970, Abcam, Cambridge, United Kingdom)[31]. The measurements were based on the reaction between MDA and thiobarbituric acid (TBA) [32]. All reagents and standard solutions were prepared according to the manufacturer's instructions. Plasma samples (20 µl) were mixed with 500 µL of 42 mM H_2SO_4, 125 µL of phosphotungstic acid solution was added, and each sample was mixed by vortexing and incubated at room temperature for 5 minutes. After incubation, we centrifuged the samples at 13000 × g for 3 minutes. The pellet was collected and resuspended in 100 µL of dH_2O on ice with 2 µL of butylated hydroxytoluene (BHT) (100-fold dilution). The final volume was adjusted to 200 µL with dH_2O. After sample preparation, the assay was performed. The MDA-TBA adduct was generated in each sample and standard solution. The TBA reagents (600 µl) were added to the samples and standards for incubation at 95 °C for 60 minutes. The reaction mixes were cooled in an ice bath for 10 minutes after incubation. The samples and standards were placed in duplicate in a 96-well microplate for analysis. The absorbance of the MDA-TBA adduct was measured at 532 nm, and the calculated concentrations are expressed in nmol/ml.

Vitamin E

A competitive enzyme immunoassay technique was used to determine the plasma vitamin E concentration (E12V0032, BlueGene Biotech., Shanghai, China). The reagents were prepared as described in the manual. First, 100 µl of plasma, standard and blank were added to a microtiter plate, each in duplicate. The plate was incubated for 90 minutes at 37 °C. After incubation, a washing step was performed two times. Then, 100 µl of vitamin E antibody was

added to each well, and the plate was incubated again for 60 minutes at 37 °C. After incubation, the contents of the wells were washed three times, and enzyme conjugate (100 µl) was added to each well. The plate was covered and incubated for 30 minutes at 37 °C. Then, the contents of the ELISA plate were washed five times, and 100 µl of color reagent liquid was added to each well. The plate was incubated for 12 minutes (the protocol mentioned that the incubation time should be controlled within 30 minutes) at 37 °C, and 100 µl of color reagent C was pipetted into each well. The absorbance values were measured at 450 nm, and the vitamin E concentrations are expressed in µg/ml.

Vitamin C

To determine the vitamin C concentration, a plasma-specific assay kit was used (ab65656, Abcam, Cambridge, United Kingdom)[33–35].The measurements were performed according to the protocol in the manual with some modifications. The kit's supernatant assay buffer was set to pH 7.0 with NaOH (10 M). The kit provides a sensitive method for vitamin C measurement. Plasma vitamin C reduces Fe^{3+} to Fe^{2+}, which shows strong absorbance that can be monitored between 545-600 nm. The reagent and standard were prepared as described in the protocol. Fifty microliters of each plasma sample, run in duplicate, was diluted two fold for measurement. The assay procedure was followed as described in the manual. The absorbance of each sample was measured at 593 nm, and the concentrations were calculated as recommended and are expressed in nmol/ml.

Reduced glutathione

The GSH concentration was measured by the enzymatic recycling method using a specific assay kit(703002,Cayman Chemical Company, Ann Arbor, MI, United States)[36–38].This assay is based on the reaction between5,5'-dithio-bis (2-nitrobenzoic acid) (DTNB) and GSH and generates a yellow product (2-nitro-5-thiobenzoic acid). All of the reagents and standards were prepared as described in the "reagent preparation" and "standard preparation" sections.

The assay was performed following the protocol in the manual. We used 50 µl plasma samples for the measurements. The concentration of GSH was determined by measuring the absorbance at 405 nm. The concentration of GSH in each sample was determined by comparison with a GSH standard curve and is expressed in µM.

Glutathione peroxidase

The activity of GPx was measured with a commercially available kit(ab102530, Abcam, Cambridge, United Kingdom)[39–43]. In the assay, GSSG produced after GPx oxidizes GSH during H_2O_2 reduction. GSSG is reduced back to GSH by GR with the aid of nicotinamide adenine dinucleotide phosphate (NADPH). The reduction of NADPH is proportional to GPx activity and can be measured colorimetrically at 340 nm. The kit reagents were dissolved as described in the manual. A standard curve was prepared as described in the kit [44].Measurements were performed according to a previous study [45]. The activity of GPx is expressed as mU/ml.

Glutathione reductase

The activity of GR in plasma was determined using a specific assay kit(ab83461, Abcam, Cambridge, United Kingdom)[46].In this assay, GSH is formed from GSSG by GR; then, GSH reacts with DTNB, and a 2-nitro-5-thiobenzoate anion (TNB^{2-}) is generated[44]. The change in absorbance was measured at 405 nm. The kit reagents were dissolved as described in the "components and storage" section. The protocol recommends pretreating the samples. First, 5 µL of 3% H_2O_2 was added to 100 µL of each sample. The samples were incubated at 25 °C for 5 min. Then, 5 µL of catalase was added to each sample, and we incubated the samples again at 25 °C for another 5 min. After the pretreatment procedure, 50 µL of each pretreated sample was added to the sample wells. The standard curve was prepared as described in the manual, and 50 µL of diluted standard solution was added to each well. Measurements were performed according to a previous study[45]. GR activity is expressed in nmol/min/mL = mU/ml.

SOD inhibition rate

The superoxide dismutase(SOD)inhibition rate was measured with a specific assay kit(ab65354, Abcam, Cambridge, United Kingdom)[47–50].In this assay, xanthine oxidase produced superoxide anions, and the conversion of superoxide anions into hydrogen peroxide was catalyzed by SOD. Superoxide anions and the water-soluble tetrazolium salt WST-1 can react to produce a water-soluble formazan dye, the absorbance of which was detected at 450 nm. The reagents were prepared as described in the kit. First, 20 µL of Blank 1, Blank 2, Blank 3 or sample was added to a 96-well plate in duplicate. Then, WST-1 solution (200 µl) was added to each blank and sample. Twenty microliters of dilution buffer were added to the Blank 2 and Blank 3 solutions. Enzyme working solution (20 µl) was added to each sample and Blank. Then,the plate was incubated at 37 °C for 20 minutes. The absorbance (A) was then measured at 450 nm. We used the following equation to calculate the SOD inhibition rate:

$$SOD\ inthibitation\ rate\ (\%) = \frac{(A_{blank\ 1} - A_{blank\ 3}) - (A_{sample} - A_{blank\ 2})}{(A_{blank1} - A_{blank\ 3})} * 100$$

Free thiol concentration

The free thiol concentration was determined through Ellman's assay (Ethos Biosciences Inc., Philadelphia, PA, United States). The kit is based on the reaction between DTNB and disulfide bonds to produce 2-nitro-5-thiobenzoic acid, and the absorbance of this product can be measured at 405 nm[51–54].The reagents and standard were prepared as recommended in the manual. Fifty microliters of each sample (15-fold dilution) or standard was added into the appropriate well in duplicate. Then, 150 µl of Ellman's reagent was pipetted into each well. The absorbance was measured immediately at 405 nm. The concentration of free thiols in each sample was determined by comparing the absorbance values of the samples to a standard curve. The free thiol concentration is expressed as the DTNB-thiol concentration in µM.

Albumin

The albumin concentration was determined using a commercially available reagent (4125S, Diagnosticum Zrt., Budapest, Hungary). The protocol was based on the method of Doumas et al. (1971)[55]. Plasma samples (3 µl/sample) and working reagent (300 µl) were mixed and incubated in a microplate at 37 °C for 3 min. Then, the absorbance was measured at 628 nm. The following equation was used to calculate the albumin concentration:

$$Albumin\ (g/l) = \frac{A_{sample}}{A_{standard}} * concentration\ of\ the\ standard$$

The abovementioned parameters were measured colorimetrically with a spectrophotometer (Spentrophotostar^{nano}, BMG Labtech). The results were calculated using MARS data analysis software.

Determination of aspartate aminotransferase (AST) levels

Plasma samples (200 µl) were pipetted into Hitachi cups (10394246001) and assessed using ASTL reagent (20764949322, ASTL 500T Cobas C Integra) Both the cup and ASTL reagent were from ROCHE Ltd. (Budapest, Hungary). The AST level was determined by measuring the decrease in absorbance using Cobas C 311 instrument (ROCHE Ltd., Budapest, Hungary).

Determination of total thiol and disulfide contents

Xiaoyun Fu et al. [56] developed a new method for thiol and disulfide content determination, which was used here. Samples were prepared according to the methods for cysteine (Cys), homocysteine (Hcy), cysteinyl-glycine (Cys-Gly), γ-glutamylcysteine (γ-GC), cystine (CySS), glutathione (GSH) and N-acetylcysteine (NAC).

Preparation of standard solutions

A standard mixture (10 µg/ml for each compound) was prepared from the Cys, Hcy, Cys-Gly, γ-GC, CySS, and GSH standards (168149-25g, 69453-10mg, C01666-25mg, G0903-

25mg, C8630-1g, and PHR1359-500mg; Merck Life Science Ltd., Budapest, Hungary). NAC (A15409.14) was purchased from VWR International Ltd., Debrecen, Hungary.

To 150 µl of standard mixture were added 300 µl of N-ethylmaleimide (NEM) solution (100 µg/ml), 1020 µl of water and 30 µl of formic acid solution (0.01% V/V). The reaction mixture was incubatedat 37 °C for 30 minutes. After cooling, standard solutions were prepared at seven concentrations (0.1, 1, 5, 10, 25, 50 and 100 ng/ml) by diluting with water.

LC–MS analysis

Analyses were performed using a Dionex Ultimate 3000RS UHPLC system (Thermo Fisher, Waltham, MA, USA) coupled to a Thermo Q Exactive Orbitrap hybrid mass spectrometer equipped with an Acclaim Mixed-Mode HILIC-1analytical column (2.1 × 150 mm, 3 µm particle size). The flow rate was maintained at 0.3 mL/min. The column oven and post column cooler temperatures were set to 25 °C ± 1 °C. The temperature of the samples was 25 °C ± 1 °C. The mobile phase consisted of water (A) and methanol (B) (both acidified with 0.1% formic acid). The gradient elution program was as follows: 0-1 min, 95% A; 1–6 min, →0% A; 6–10 min, 0% A; 10–10.5 min, →95% A; 10.5-20 min, 95% A. The injection volume was 5 µL.

The Thermo Q Exactive Orbitrap hybrid mass spectrometer (Thermo Fisher, Waltham, MA, USA) was equipped with a HESI source. The samples were analyzed in positive ion mode using the selected ion monitoring (SIM) technique with the following inclusion list ($[M + H]^+$): cysteine-NEM ($C_9H_{15}N_2O_4S$): 247.07525; N-acetylcysteine-NEM ($C_{11}H_{17}N_2O_5S$): 289.08582; homocysteine-NEM ($C_{11}H_{17}N_2O_5S$): 261.09090; cysteinylglycine-NEM ($C_{11}H_{18}N_3O_5S$): 304.09672; γ-glutamylcysteine-NEM ($C_{14}H_{22}N_3O_7S$): 376.11785; cystine ($C_6H_{13}N_2O_4S_2$): 241.03167; glutathione-NEM ($C_{16}H_{25}N_4O_8S$): 433.13931; and glutathione disulfide ($C_{20}H_{33}N_6O_{12}S_2$): 613.15979.

The capillary temperature of the mass spectrometer was set to 320 °C, the spray voltage was 4.0 kV and the resolution of 35,000. The sheath gas flow rate and aux gas flow rate were 32 AU and 7 AU, respectively. The differences between the measured and calculated monoisotopic molecular masses were less than 5 ppm in each case. The data were acquired and processed using Thermo Xcalibur 4.0 software (ThermoFisher, Waltham, MA, USA).

Determination of cytokine concentrations

The concentration of interleukin 2 (IL-2) was determined using a commercially available sandwich ELISA kit (E0003Ch, Bioassay Technology Laboratory, Junjiang International Bldg., Shanghai China). In this ELISA, the plate was pre coated with chicken IL-2 antibody, and the sample was added so that the IL-2 in the sample could bind to the antibodies coated on the wells. The reagents were prepared as described in the manual. Fifty microliters of standard solutions and 40 µl of sample were added into the appropriate wells. IL-2 antibody (10 µl) was pipetted into the sample wells, and 50 µl of streptavidin-HRP was added to the sample wells and standard wells (but not the blank control wells). The plate was covered and incubated for 60 minutes at 37 °C. After incubation, the cover was removed, and the contents of the plate were washed 5 times with wash buffer (350 µl); each washing step took 1 minute. Fifty microliters of substrate solution A and 50 µl of substrate B were added to each well. The plate was sealed and incubated for 10 minutes at 37 °C in the dark. Then, stop solution (50 µl) was pipetted into each well, and the blue color immediately changed to yellow. The optical density (OD value) of each well was determined immediately at 450 nm using a microplate reader (within 10 minutes after adding the stop solution). The free IL-2 concentration is reported as ng/l.

The levels of interleukin 6 (IL-6), interleukin 8 (IL-8) and tumor necrosis factor-α were determined using commercially available sandwich ELISA kits (E0004Ch, E0005Ch, and E0025Ch) from Bioassay Technology Laboratory, Junjiang International Bldg.,Shanghai

China. All of the ELISA kit principles and assay procedures were the same as those of the IL-2 ELISA kit.

Histological examinations

Formalin-fixed liver tissue samples were embedded in paraffin. Four-micron-thick sections were generated with a rotary microtome and routinely stained with hematoxylin and eosin[57].

Statistical analysis

Statistical analyses and data visualization were performed using GraphPad Prism (version 9). The antioxidant parameters and concentrations of total thiols, disulfides and cytokines were analyzed by one-way analysis of variance (ANOVA) followed by Tukey's method for multiple comparisons. A value of $p<0.05$was considered to indicate statistical significance, and the results are presented as the mean ± SEM. The correlation between biochemical parameters was analyzed by Pearson's correlation ($p< 0.05$).

Results and Discussion

Growth Performance

During the four phases of feeding, the body weight of the birds was 232 g on the 9th day, which tripled on Day 21. A further significant increase in weight occurred during the 3rd stage of growth. By the end of the experiment, the average weight of the birds was 2758 g. Dietary indicators related to growth performance, such as body weight (BW), average daily gain (ADG), average daily feed intake (ADFI) and feed conversion ratio (FCR), were measured (Table 2).

The mortality of the birds was monitored and found to be very low (0.56%), and there was no association between mortality and nutrition. No veterinary treatment was required for the entire duration of the experiment.

The results of the current study indicate that the performance of the experimental subjects corresponds to the values typical of fast-growing broilers. The data also show that the performance of broilers did not indicate the presence of metabolic diseases among the livestock**[**58–61**]**.

Table 2. Growth performance of the broiler chickens

	BW (g)	*ADG (g/day/bird)*	*ADFI (g/day/bird)*	*FCR (kg feed/kg BW)*
**Prestarter (Days 1-9)**	232	19	4	0.19
**Starter (Days 10-21)**	759	~~48~~	50	1.35
**Grower (Days 22-31)**	1713	87	130	1.23
**Finisher (Days 32-42)**	2758	104	114	1.26

**Entire experiment (Days 1-42)**	2719	65	73	1.06

[a]Mean values. BW and ADG are based on individual values (n = 18).The ADFI was calculated per pen (n = 3)

Redox parameters

Genetic selection of broilers facilitates animal susceptibility to biotic and abiotic stress factors. Oxidative stress is known to be one of the most important stressors and can be accompanied by serious outcomes; thus, it is among the most studied parameters[1,2]. Oxidized thiol compounds can be considered reactive oxygen species (ROS) that affect cellular calcium (Ca^{2+}) homeostasis, which in turn leads to structural damage to Ca^{2+} ATPase. Accumulation of Ca^{2+} can cause damage to the endoplasmic reticulum (ER), which might induce multiple pathological alterations [62,63].

The impact of oxidative stress on physiological functions has received exceptional scientific interest for decades. Its characteristic parameters, namely, (i) enzyme activities (SOD, catalase, glutathione-S-transferase, GR and GPx), (ii) concentrations of small antioxidant molecules (vitamins E and C and GSH), and (iii) the expression of heat shock proteins, enable us to study the role of a specific substance or environmental factor in the maintenance of redox homeostasis [64,65]. However, the dynamic characteristics of redox homeostasis should be taken into consideration, meaning that accurate conclusions cannot be drawn regarding the oxidant/pro-oxidant balance and pathological conditions from measurements taken at only one-time point. The status of redox homeostasis must be followed during the whole life cycle, which makes it possible to compare different developmental stages.

In samples taken at the first time point, the plasma MDA concentration was 187 nmol/ml (Fig. 1A). In the plasma of 21-day-old animals, this value had decreased, but then it increased significantly until the end of the experimental period. In contrast, the blood plasma vitamin E concentration was the highest on Day 21 (26.6 ng/ml), but by the end of the experiment (Day 42), it decreased (Fig. 1B). As the MDA concentration increased, the amount of α-tocopherol decreased. This is not surprising because if the contents of pro-oxidants, which cannot be eliminated by the redox system, increase, the hydroxyl radicals from the Fenton reaction can

react with lipid molecules and give MDA[66–68]. Tocopherol behaves as a H^+ donor and transforms into α-tocopherol radicals after reaction with OH^-radicals, which are regenerated by vitamin C[69–71].Therefore, we can conclude that the antioxidant status of the animals declined and the number of lipid peroxidation processes increased.

Similar to that of MDA, the blood plasma vitamin C concentration was the lowest on Day 21 and then increased significantly by the end of the experiment it increased significantly($p<0.000001$) (Fig. 1C). As the amount of free radicals increases, the body is supposed to eliminate them, and in turn, the synthesis of ascorbic acid increases in the liver. The α-tocopherol radical is transformed into α-tocopherol and inhibits lipid peroxidation processes[72,73]. Thus, it can be concluded from our results that the elevation of the antioxidant level was not neutralized by the extensive synthesis of pro-oxidants.

Fig. 1. Changes in the redox parameters in blood plasma at Days 3, 8, 21, 32, and 42.

(A) MDA concentration. (B) Vitamin E concentration. (C) Vitamin C concentration. (D) GSH concentration. (E) GPx activity. (F) GR activity. (G) SOD inhibition rate. (H) DTNB-thiol concentration. (I) Albumin concentration. Significant differences were determined by comparing the data from each time point with the data from the first time point. Data are expressed as the means ±8 SEMs; *$P<0.05$, ** $p<0.005$, *** $p<0.001$.

The GSH concentration showed a continuous, significant ($p<0.000001$) decrease throughout the experiment (Fig. 1D). Hydrogen peroxide (H_2O_2) was eliminated by GPx using GSH. GSH is the most important small molecule antioxidant in the body, which eliminates pathological free radicals and lipid peroxides. GSH has a significant role in the redox cycle of cells[74,75].

In birds, this enzyme has an important role in the maintenance of redox homeostasis. In spite of the decreasing GSH level, by breaking down the large amount of glutathione disulfide

(GSSG) produced, GR contributes to the maintenance of the activity of glutathione-dependent GPx. GPx activity significantly decreased by Day 21 ($p<0.000001$) and then significantly increased until the end of the experiment ($p<0.000001$) (Fig. 1E).

The GSH level decreased although the activity of GR, which cleaves conjugated glutathione, showed a continuous significant increase ($p=0.000036$) during the 42 days (Fig. 1F).

GSH is structurally a tripeptide (Gly-Cys-Glu)[76]that protects enzymes with thiol groups, e.g., GR [EC 1.6.4.2] and GPx [EC 1.1.1.9], from inactivation. It is a cofactor of GPx [77] and a strong reducing agent that is oxidized during these reactions, while oxidized glutathione forms through the SH-bridge. In the reaction catalyzed by the NADPH-dependent GR, oxidized glutathione is converted back into GSH at a specific rate (the characteristic rate of the reduced to oxidized form in a healthy body is 500:1)[73,76,77]. The glutathione concentration decreased by almost half even with intensive GR in 21-day-old birds, indicating significant oxidative stress (Fig. 1). This reduction in GPx activity had adverse effects on the regeneration and detoxification pathways, implying an increase in the vitamin E blood plasma concentration. However, lipid peroxidation was not compensated for, resulting in elevated MDA levels [80,81]. This raises the issues of when GSH diminishes and which biochemical processes are able to relieve the body of the abundant pro-oxidants.

The SOD [EC 1.15.1.1] inhibition rate significantly decreased ($p<0.000001$) by Day 21 and slightly increased ($p<0.000001$) by Day 32 (Fig. 1G), and an additional decrease was observed at the end of the experimental period ($p<0.000001$). The plasma free thiol concentration significantly decreased by Day 21 ($p<0.00001$), and then a further decrease was observed on Day 32. By the end of the experiment, a significant increase was measured (Fig. 1H). A significant increase was detected in albumin concentration until Day 42 ($p<0.000001$) (Fig. 1I).

According to the latest research, large molecule antioxidant proteins are responsible for the antioxidant status of plasma[82]. In the blood plasma of birds, albumin is found in the largest concentration (average 12-15 g/l)[83]. During development and growth, albumin levels are stable in plasma, and significant changes might lead to fatal consequences[84–86]. However, during certain periods, significant differences can be seen[83], and such changes can be explained by the rhythm of albumin exchange. The degree of freedom of the Cys binding site is responsible for the antioxidant properties of albumin[87–89].

Although the antioxidant capacity of albumin is generally not studied in animals, clinical data are already available regarding its role in the maintenance of redox homeostasis since it is the most abundant protein in the blood plasma. Medical use of human serum albumin (HSA) has been widely spread in critical cases (sepsis and cirrhosis) for a long time. HSA can not only maintain osmotic pressure but also has special antioxidant functions derived from its ability to scavenge free radicals[90]. Its principal ligands, intermediate ionic forms of copper (Cu^{2+}) and iron (Fe^{2+}), are implicated in H_2O_2 elimination (Haber-Boss and Fenton reactions); thus, has works as an inhibitor of lipid peroxidation [91,92]. Moreover, because of its free Cys34 structure, it can reversibly bind GSH and Hcy, forming mixed disulfides [93]. Approximately two-thirds of HSA is in its reduced form mercaptoalbumin, while one-third is in the mixed disulfide form, in which Cys can bind superoxide anions and be oxidized to the stable sulfenic acid form (HSA-SOH). Therefore, HAS has gained increasing attention in poultry [94]Complex structural analysis of chicken serum albumin (CSA) has determined that Cys has the same function in CSA as in HSA. The position of Cys in proteins is highly conserved, and Cys can generally be found in functional or structural regions responsible for catalytic metal binding and/or redox regulating activity[95,96]. Our investigation of the saturation of CSA during the whole lifecycle revealed that the free binding site of CSA was saturated owing to oxidative

stress on Day 21. CSA capacity analyses might provide data regarding redox homeostasis and the health status of animals.

The concentration of thiols

The total plasma concentration of thiols and disulfides (TTD) is shown in Fig. 5. At every time point, the participants of Cys homeostasis and TSP were examined.

During the TTD measurements, we focused on the participants in Cys homeostasis and TSP (Fig. 2). The results from the thiol derivatives corresponded well with the alterations in antioxidant parameters. Changes in GSH concentration showed similar results as those from the colorimetric assay (Fig. 2B). The most salient decrease in GSH levels was detected on Day 21 ($p<0.000001$), which slightly decreased further until the end of the experimental period. This was in contrast with the γ-GC concentration, which is the precursor of GSH **[**97**]**. The concentration of γ-GC increased by Day 8 and remained almost unchanged thereafter (Fig. 2F).

Significant changes in the concentrations of GSH and CySS, the oxidized dimer form of Cys[98], corresponded well with the alterations in the disulfide Cys-Gly level (Fig. 5B, 5F, 5G).Cys-Gly is formed during the degradation of GSH and CySS [99]. The amounts of GSH and CySS decreased after a solid initial rise, while that of Cys-Gly decreased at first and then increased.

Cys, one of the components of GSH, is known to be an important structural and functional element of proteins because of its free thiol group.[100]In the extracellular space, its free form is very reactive, which in turn can cause the generation of toxic free radicals. [101,102]Considering that protein-bound Cys is an important indicator of antioxidant status, it can be characterized well by estimating the bound and total Cys levels. Moreover, the GSH content in plasma is generally low, and GSH eliminates free radicals only intracellularly; thus, GSH content cannot provide enough information about antioxidant status[103,104]. Our

measurements showed a significant elevation in the Cys level in fast-growing broilers on Day 8, and the Cys level remained high during the whole lifecycle of the birds (Fig. 2A).

Hcy is an analog of Cys, which is formed from methionine and can be transformed into Cys in the TSP or reverted to methionine after remethylation [99,105]. The Hcy level was elevated by more than two fold on Day 8 and remained high until Day 42 (Fig. 2D).

The concentration of NAC changes significantly during certain growth periods, and a continuous decrease was observed compared to that on the 3rd day.

The NAC measurement results are not surprising either since albumin saturation is very high in 21-day-old animals. One of the tasks of NAC is to breakdown the mixed disulfides of human serum albumin (HSA) and thereby increase the degree of freedom at the Cys34 binding site[106,107]

Fig. 2. The concentration of total thiols in the blood plasma on Days 3, 8, 21, 32, and 42. (A) Cys concentration. (B) GSH concentration. (C) NAC concentration. (D) Hcy concentration. (E) Cys-Gly concentration. (F) γ-GC concentration. (G) Significant differences in CySS were detected when comparing the data from the first time point with the data from each subsequent time point. Data are expressed as the means ± SEMs; *$P<0.05$, ** $p<0.005$, *** $p<0.001$.

The crucial roles of free and bound thiols has already been proven in human studies, but there is not yet a suitable method for their measurement. A novel method published by Xiaoyun Fu et al.[108] provides a reliable and reproducible procedure to analyze all types of thiol compounds.

Thiols fulfil a prominent role in the three-level antioxidant protection system: (1) the reducing agent GSH, which is detectable in the extracellular space in lower amounts; (2) the regulation of redox status; and (3) immunological functions [109]. GSH is generated from

CySS/Cys by the rate-limiting enzymes γ-GC synthetase (γ-GCS) and glutathione synthetase (GSS). In addition to being a precursor in GSH synthesis, cystine/Cys is a critical substrate of protein synthesis as well as an extracellular reducing agent. In addition, precursors of Hcy are produced via the TSP[99,110].

GSH degradation is catalyzed by γ-glutamyl transpeptidase (γ-GT) to generate a Cys-Gly and a glutamic acid (Glu) residue exclusively in the extracellular space of mammalian cells. Glu is transferred to another acceptor amino acid, Cys-Gly, which is degraded further by a dipeptidase [111] These processes demonstrate that GSH homeostasis is a combination of its intracellular synthesis and utilization as well as export and extracellular degradation. We were able to detect all of the abovementioned components in the blood plasma of fast-growing broilers (Fig. 2)[112–115].

Thiols are present in the cells in millimolar concentrations mainly in their reduced forms. The total glutathione concentration is 2-17 mM, 91% of which is GSH[116,117]. In contrast, in the extracellular space and especially the plasma, thiols are rapidly oxidized, resulting in a lower concentration, 0.4-0.6 mM, in the blood plasma. The concentrations of small thiol molecules, such as Cys, Cys-Gly, GSH, Hcy and γ-GC, are12-20 µM, and the total GSH content is 50-55%. In addition, Hcy can form disulfide bridges and be produced from thiols and might modify lysine residues, which in turn can lead to N-homocysteine residues in peptides[118].

The measurement results suggest that there is a close connection between the defense system that maintains redox homeostasis and the TSP (Fig 3)[119]. In this system, the measured change in Hcy concentration can be of diagnostic importance, as it predicts the emerging GSH deficiency, the increase in the expression level of inflammatory cytokines, and the development of nonalcoholic fatty liver disease[120,121].The consequence of liver tissue damage is the increased concentration of necroenzymes in the blood[122,123].Another consequence of damaged liver tissue is that it cannot express the enzymes cystathionine β-synthase (CBS) and

cystathionine γ-lyase (CTH), which are responsible for breaking down Hcy [99].This has further consequences, e.g., there is a lack of H_2S, which results in the redox balance being disturbed, which generates new inflammatory processes through the NF-κB pathway[124,125].

Fig. 3. Transsulfuration pathway and antioxidant system.

Tetrahydrofolate (THF); 5,10-methylene-tetrahydrofolate (5,10-Methylene-THF); methionine synthase (MS); methionine (Met);betaine homocysteine methyltransferase (BHMT); dimethylglycine (DMG); S-adenosylmethionine (SAM); methionine adenosyltransferase (MAT); S—adenosylmethionine (AdoMet); glycine N-methyltransferase (GNMT); S-adenosylhomocysteine (AdoHcy); S-adenosylhomocysteine hydrolase (SAHH); homocysteine (Hcy); cystathionine β-synthase (CBS); cystathionine γ-lyase (CTH); α-ketobutyrate (αKB); aspartate aminotransferase (AAT); sulfinoalanine decarboxylase (SAD); Hypotaurine dehydrogenase (HPD); cystine reductase (Cyss R); Glutathione-cystine transhydrogenase (GSH-Cyss TH); γ-glutamylcysteine synthase (GCS); glutathione synthase (GS); and aspartateaminotransferase(AST)

Relationship between the antioxidant system and inflammatory markers

Cytokine levels increased in parallel with the weakening antioxidant capacity as the birds grew (Fig. 4). We observed an approximately fourfold increase in all parameters from Day 21.

IL-2, IL-6, IL-8 and TNF-α have crucial roles in stimulating, proliferating and differentiating T-helper cells, B cells and natural killer cells as well as in the recruitment of other immune cells**[**126–128**]**. Our results also showed that the concentrations of these markers were extremely high on Day 42, which denotes severe inflammation in the animals. However, the most appropriate marker and time point for measurement to evaluate inflammatory status as accurately as possible is still elusive.

Fig. 4. The concentrations of inflammatory markers in the plasma at Days 3, 8, 21, 32, and 42.

(A)IL-2 concentration. (B) IL-4concentration. (C) IL-6concentration. (D) TNF-α concentration. Significant differences were determined by comparing the data from each time point with the data from the first time point. Data are expressed as the means ± SEMs; $*P<0.05$, $** p<0.005$, $*** p<0.001$.

The transcription factor nuclear factor κB (NF-κB) is involved in inflammation, the stress response, and cell differentiation, proliferation and apoptosis. Moreover, it regulates the gene expression of pro-inflammatory cytokines, chemokines, adhesion molecules, receptors and microRNA[129,130]. Oxidative stress and intracellular redox status have been proven to be essential in NF-κB activation; H_2O_2 can enhance, whereas antioxidants block, this process [131].

The extracellular Cys/CySS redox potential in blood plasma exposed to oxidative stress has been shown to evoke monocyte adhesion to vascular endothelial cells, activate NF-κB and enhance inflammatory cytokine expression [132,133]. In particular, interleukin 1β (IL-1β) and tumor necrosis factor alpha (TNF-α) induce the expression of their own and other cytokines. The identification of the primary disorder needs to be mentioned here. Oxidative stress that develops first in one organ leads to inflammation and enhances oxidative stress. In contrast, if inflammation is the primary disorder, it promotes oxidative stress and further increases inflammatory processes. Our data demonstrated upregulated proinflammatory cytokine expression in birds on the 21st day, which indicates the evolution of strong inflammation [134].

Reactive oxygen species (ROS) target thiol compounds of specific proteins that participate in signaling pathways implicated in cell proliferation, differentiation and apoptosis (kinases, phosphatases, and transcription factors). Moreover, hepatocytes are damaged, and ROS indicate their apoptosis, which is known to be important, especially in nonalcoholic steatohepatitis (NASH), where ROS are created in multiple ways [135–137].

ROS production and mitochondrial dysfunction are a result of complex metabolic processes, including enhanced β-oxidation and inhibition of the mitochondrial electron transport chain by TNF-α and lipid peroxidation products. Mitochondrial damage results in the secondary inhibition of β lipids, and oxidation increases steatosis levels[138].

Oxidative stress and antioxidant protection have also been detected in fibrosis and cirrhosis in clinical and animal studies. DNA lipids and proteins disintegrated by oxidative stress lead to necrosis, the loss of hepatocytes and an escalating inflammatory response, which in turn can cause fibrosis [139]. Inflammation evoked by necrosis in the priming phase of hepatic impairment contributes to removing cellular debris and promoting liver regeneration, ensuring restoration of the liver structure and function following acute damage[139,140]. Progressive liver fibrosis and chronic inflammation can develop in cases when the primary disease and sustained stimuli are not controlled, which together with necrosis, are characteristic of fibrotic liver disorders [141]. This was confirmed by our results from 21-day-old birds, namely, the level of AST, a necrotic enzyme, was increased significantly.

The plasma aspartate aminotransferase (AST) concentration significantly increased on Day 21($p<0.000001$), then on both Day 32 ($p=0.000009$) and Day 42 ($p=0.000003$) compared to the initial day (Fig. 5). After Day21, it moderately decreased but remained very high until Day 42.

Fig. 5. The concentration of AST in plasma at Days 3, 8, 21, 32, and 42.
Significant differences were detected when comparing the data from the first time point with the data from subsequent time points. Data are expressed as the means ± SEMs; *P<0.05, ** p<0.005, *** p<0.001.

Adaptive immune responses provoked by oxidative stress might be independent predictors of fibrotic alterations in nonalcoholic fatty liver disease (NAFLD) [142]. The

pathogenesis of NAFLD is extremely complex and accompanied by modified molecular biological and metabolic pathways. The TPS is involved in these events and is correlated with steatosis, insulin resistance, oxidative stress, ER stress, inflammation and portal hypertension[99]. The correlation between the TSP and oxidative stress has already been proven since TSP is a regulator of GSH production. Elevated plasma Hcy levels, steatosis, oxidative stress and fibrosis were found in mice with cystathionine beta synthase (CBS) deficiency[143,144]. The expression of genes implicated in ER stress, liver lipid homeostasis and steatosis seems to be upregulated by CBS deficiency, while knockout of cystathionine gamma synthase (CSE) causes a decrease in liver lipolysis [142,145,146].

TSP coverts Hcy into Cys via the intermediate cystathionine and has a key role in sulfur metabolism and the cellular redox system, which is considered to be the only pathway of Cys biosynthesis. The first step is catalyzed by vitamin B6-dependent CBS to generate cystathionine via a condensation reaction using Hcy and serine as substrates. Cystathionine is then hydrolyzed by vitamin B6-dependent CSE to form Cys and α-ketobutyrate (aKB)[99].

It is important to note that Hcy-Cys conversion is irreversible. Therefore, all downstream pathways of TSP are part of Cys metabolism. The nonessential amino acid Cys is a crucial participant in sulfur metabolism and a precursor of significant metabolites, including GSH, H_2S, sulfate and taurine. A lack of these molecules has been proven to generate oxidative stress and ultimately induce inflammation. Damage to hepatocytes downregulates the expression of the Hcy-degrading enzymes CBS and CSE, and the accumulation of Hcy hampers the glutathione pool, which finally creates a vicious cycle[99,110,147].

Correlation between thiol compounds, cytokines and antioxidant parameters

The enzymes responsible for Hcy degradation outside the liver are expressed in endothelial cells in the wall of the aorta and in the brain. The resulting systemic diseases are likely to be related to elevated Hcy concentrations.

The relationships between the measured antioxidant parameters were determined by correlation analysis. The correlations are illustrated on a heatmap with a gradient of colors, where the red scale represents negative correlations and the blue scale represents positive correlations (Fig. 7). The concentrations of total cysteinylglycine, IL-6, IL-8 and TNFα increased in parallel with the increase in MDA concentration. The increased activity of GR is negatively correlated with the concentration of CySS. This shows that the consequence of the increase in lipid peroxidation processes is the decomposition of GSH, since the concentration of the decomposition product increases but the amount of the precursor, which is indispensable for its synthesis, decreases. This helps with the initiation of inflammatory processes[148–150].Presumably, the body tries to protect itself against damage to the membrane system by synthesizing vitamin C[151].In the case of GSH, we observed correlations that were contrary to this since the activity of GR decreased in the presence of GSH. Moreover, since a suitable reduced form is available in the defense system, SOD activity is high, albumin saturation is low, and there are many forms of free thiols in plasma. GSH levels are also negatively correlated with the level of inflammation. If albumin saturation in the plasma is low, then the concentrations of thiol components, including GSH, is high, so the redox system is not damaged and inflammatory cytokines are expressed to a lesser extent[152].

Fig. 6 Correlations betweenantioxidant parameters, cytokines and thiols.

*, **, *** indicate significant differences at $P < 0.05$, $P < 0.01$, and $P < 0.001$, respectively.

Histological alterations tothe liver

In addition to increasing levels of the main inflammatory indicators, structural alterations to the liver were clearly seen in histological sections (Fig. 8), and included hydropic degradation, the infiltration of inflammatory cells, and serious intrahepatic bleeding[153].

According to the histological image of the liver (Fig. 8), the number of lipid deposits characteristic of fatty degeneration of the liver also increased significantly in the 42-day animals. This was also reflected in the number of Mallory bodies, indicating the aggregation of damaged intermediate filaments[154,155].

Fig. 7. Representative micrographs of a chicken liver.

(A) Section taken at Day21. (B) Section taken at Day 42

Conclusion

From the present study, the following main conclusions can be drawn.

- In our study, growth performance did not differ from the typical literature values for fast-growing broilers.
- It can be seen that 21 days is critical time point for birds, and a large increase in body mass is associated with the formation of many free radicals. The animal's body maintain a balance, so the synthesis of vitamin C in the liver increases, decreasing the plasma level. The binding site of CSA was saturated owing to oxidative stress on Day 21. The antioxidant defense system cannot neutralize the intense pro-oxidant synthesis.
- The levels of thiols and pro-inflammatory cytokines in birds on the 21st day indicate the evolution of strong inflammation. There is a close connection between redox homeostasis and the TSP. According to the Hcy concentration and histological alterations, nonalcoholic fatty liver disease may developat42 days. This was evidenced by the increased lipid deposition that is characteristic of fatty degeneration of the liver.
- Monitoring changes in Hcy concentration could be of diagnostic importance.
- It is important to find the stage of grow that which strong support of the redox system in birds is needed. For this, it is necessary to look for new biomarkers to measure that are easily recognized when the animals' redox balance is disturbed.
- Although it cannot be detected visually, the birds are already sick physiologically, a conclusion that is supported by the results of the histological and clinical parameters. From an animal welfare point of view, this effect is not negligible.

Acknowledgments

We gratefully thank Gábor Méhes director of Pathology University of Debrecen, for the histological examinations. We are grateful to all the coworkers of the Kismacs Experimental Station of Animal Husbandry.

References

1. Hartcher K, Lum H. Genetic selection of broilers and welfare consequences: a review. World's Poultry Science Journal. 2019;76: 1–14. doi:10.1080/00439339.2019.1680025

2. Baghbanzadeh A, Decuypere E. Ascites syndrome in broilers: physiological and nutritional perspectives. Avian Pathology. 2008;37: 117–126. doi:10.1080/03079450801902062

3. Wideman RF. Pathophysiology of heart/lung disorders: pulmonary hypertension syndrome in broiler chickens. World's Poultry Science Journal. 2001;57: 289–307. doi:10.1079/WPS20010021

4. Rowe E, Dawkins MS, Gebhardt-Henrich SG. A Systematic Review of Precision Livestock Farming in the Poultry Sector: Is Technology Focussed on Improving Bird Welfare? Animals. 2019;9: 614. doi:10.3390/ani9090614

5. Skaperda Z-V, Veskoukis A, Kouretas D. Farm animal welfare, productivity and meat quality: Interrelation with redox status regulation and antioxidant supplementation as a nutritional intervention (Review). World Academy of Sciences Journal. 2019. doi:10.3892/wasj.2019.19

6. Lauridsen C. From oxidative stress to inflammation: redox balance and immune system. Poultry Science. 2019;98: 4240–4246. doi:10.3382/ps/pey407

7. Celi P, Gabai G. Oxidant/Antioxidant Balance in Animal Nutrition and Health: The Role of Protein Oxidation. Frontiers in Veterinary Science. 2015;2. Available: https://www.frontiersin.org/articles/10.3389/fvets.2015.00048

8. O'Reilly EL, Burchmore RJ, Sparks NH, Eckersall PD. The effect of microbial challenge on the intestinal proteome of broiler chickens. Proteome Science. 2017;15: 10. doi:10.1186/s12953-017-0118-0

9. Zhang J, Li C, Tang X, Lu Q, Sa R, Zhang H. Proteome changes in the small intestinal mucosa of broilers (Gallus gallus) induced by high concentrations of atmospheric ammonia. Proteome Sci. 2015;13: 9. doi:10.1186/s12953-015-0067-4

10. Lake JA, Dekkers JCM, Abasht B. Genetic basis and identification of candidate genes for wooden breast and white striping in commercial broiler chickens. Sci Rep. 2021;11: 6785. doi:10.1038/s41598-021-86176-4

11. Liu R, Kong F, Xing S, He Z, Bai L, Sun J, et al. Dominant changes in the breast muscle lipid profiles of broiler chickens with wooden breast syndrome revealed by lipidomics analyses. Journal of Animal Science and Biotechnology. 2022;13: 93. doi:10.1186/s40104-022-00743-x

12. Agyare C, Boamah VE, Osei CNZ and FB, Agyare C, Boamah VE, Osei CNZ and FB. Antibiotic Use in Poultry Production and Its Effects on Bacterial Resistance. Antimicrobial Resistance - A Global Threat. IntechOpen; 2018. doi:10.5772/intechopen.79371

13. Sihvo H-K, Immonen K, Puolanne E. Myodegeneration With Fibrosis and Regeneration in the Pectoralis Major Muscle of Broilers. Vet Pathol. 2014;51: 619–623. doi:10.1177/0300985813497488

14. Liu J, Puolanne E, Schwartzkopf M, Arner A. Altered Sarcomeric Structure and Function in Woody Breast Myopathy of Avian Pectoralis Major Muscle. Frontiers in Physiology. 2020;11. Available: https://www.frontiersin.org/articles/10.3389/fphys.2020.00287

15. Mutryn MF, Brannick EM, Fu W, Lee WR, Abasht B. Characterization of a novel chicken muscle disorder through differential gene expression and pathway analysis using RNA-sequencing. BMC Genomics. 2015;16: 399. doi:10.1186/s12864-015-1623-0

16. Soglia F, Gao J, Mazzoni M, Puolanne E, Cavani C, Petracci M, et al. Superficial and deep changes of histology, texture and particle size distribution in broiler wooden breast muscle during refrigerated storage. Poultry Science. 2017;96: 3465–3472. doi:10.3382/ps/pex115

17. Xing T, Pan X, Zhang L, Gao F. Hepatic Oxidative Stress, Apoptosis, and Inflammation in Broiler Chickens With Wooden Breast Myopathy. Front Physiol. 2021;12: 659777. doi:10.3389/fphys.2021.659777

18. Maharjan P, Hilton K, Weil J, Suesuttajit N, Beitia A, Owens CM, et al. Characterizing Woody Breast Myopathy in a Meat Broiler Line by Heat Production, Microbiota, and Plasma Metabolites. Frontiers in Veterinary Science. 2020;6. Available: https://www.frontiersin.org/articles/10.3389/fvets.2019.00497

19. Bessei W. Welfare of broilers: A review. World’s Poultry Science Journal. 2006;62: 455–466. doi:10.1017/S0043933906001085

20. Kubale V, Merry K, Miller G, Rutland MRD and CS, Kubale V, Merry K, et al. Avian Cardiovascular Disease Characteristics, Causes and Genomics. Application of Genetics and Genomics in Poultry Science. IntechOpen; 2018. doi:10.5772/intechopen.78005

21. Shen M, Xie Z, Jia M, Li A, Han H, Wang T, et al. Effect of Bamboo Leaf Extract on Antioxidant Status and Cholesterol Metabolism in Broiler Chickens. Animals (Basel). 2019;9: 699. doi:10.3390/ani9090699

22. Decuypere E, Hassanzadeh M, Buys N, Buyse J. Further insights into the susceptibility of broilers to ascites. Vet J. 2005;169: 319–320. doi:10.1016/j.tvjl.2004.07.005

23. Wang Y, Guo Y, Ning D, Peng Y, Cai H, Tan J, et al. Changes of hepatic biochemical parameters and proteomics in broilers with cold-induced ascites. J Animal Sci Biotechnol. 2012;3: 41. doi:10.1186/2049-1891-3-41

24. Samuels SE. Diet, total plasma homocysteine concentrations and mortality rates in broiler chickens. Can J Anim Sci. 2003;83: 601–604. doi:10.4141/A02-029

25. Tabata F, Wada Y, Kawakami S, Miyaji K. Serum Albumin Redox States: More Than Oxidative Stress Biomarker. Antioxidants (Basel). 2021;10: 503. doi:10.3390/antiox10040503

26. Turell L, Radi R, Alvarez B. The thiol pool in human plasma: The central contribution of albumin to redox processes. Free Radic Biol Med. 2013;65: 244–253. doi:10.1016/j.freeradbiomed.2013.05.050

27. Taylor PS, Hemsworth PH, Groves PJ, Gebhardt-Henrich SG, Rault J-L. Frequent range visits further from the shed relate positively to free-range broiler chicken welfare. Animal. 2020;14: 138–149. doi:10.1017/S1751731119001514

28. Meluzzi A, Sirri F. Welfare of broiler chickens. Italian Journal of Animal Science. 2009;8: 161–173. doi:10.4081/ijas.2009.s1.161

29. Malik PK, Bhatta R, Takahashi J, Kohn R, Prasad CS. Livestock Production and Climate Change. CABI; 2015.

30. H. A. Olanrewaju, J. P. Thaxton, W. A. Dozier III, J. Purswell, W. B. Roush, S. L. Branton. A Review of Lighting Programs for Broiler Production. International Journal of Poultry Science. 5: 301–308. doi:10.3923/ijps.2006.301.308

31. Alvarez-Mon MA, Ortega MA, García-Montero C, Fraile-Martinez O, Lahera G, Monserrat J, et al. Differential malondialdehyde (MDA) detection in plasma samples of patients with major depressive disorder (MDD): A potential biomarker. The Journal of International Medical Research. 2022;50. doi:10.1177/03000605221094995

32. Grotto D, Maria LS, Valentini J, Paniz C, Schmitt G, Garcia SC, et al. Importance of the lipid peroxidation biomarkers and methodological aspects FOR malondialdehyde quantification. Quím Nova. 2009;32: 169–174. doi:10.1590/S0100-40422009000100032

33. Lee H, Ahn J, Shin SS, Yoon M. Ascorbic acid inhibits visceral obesity and nonalcoholic fatty liver disease by activating peroxisome proliferator-activated receptor α in high-fat-diet-fed C57BL/6J mice. Int J Obes. 2019;43: 1620–1630. doi:10.1038/s41366-018-0212-0

34. Molnár Á, Homoki DZ, Bársony P, Kertész A, Remenyik J, Pesti-Asbóth G, et al. The Effects of Contrast between Dark- and Light-Coloured Tanks on the Growth Performance and Antioxidant Parameters of Juvenile European Perch (Perca fluviatilis). Water. 2022;14: 969. doi:10.3390/w14060969

35. Blaschke K, Ebata KT, Karimi MM, Zepeda-Martínez JA, Goyal P, Mahapatra S, et al. Vitamin C induces Tet-dependent DNA demethylation in ESCs to promote a blastocyst-like state. Nature. 2013;500: 222–226. doi:10.1038/nature12362

36. Willemsen H, Swennen Q, Everaert N, Geraert P-A, Mercier Y, Stinckens A, et al. Effects of dietary supplementation of methionine and its hydroxy analog dl-2-hydroxy-4-methylthiobutanoic acid on growth performance, plasma hormone levels, and the redox status of broiler chickens exposed to high temperatures. Poultry Science. 2011;90: 2311–2320. doi:10.3382/ps.2011-01353

37. Swennen Q, Geraert P-A, Mercier Y, Everaert N, Stinckens A, Willemsen H, et al. Effects of dietary protein content and 2-hydroxy-4-methylthiobutanoic acid or dl-methionine

supplementation on performance and oxidative status of broiler chickens. British Journal of Nutrition. 2011;106: 1845–1854. doi:10.1017/S0007114511002558

38. Russell JR, Sexten WJ, Kerley MS, Hansen SL. Relationship between antioxidant capacity, oxidative stress, and feed efficiency in beef steers1. Journal of Animal Science. 2016;94: 2942–2953. doi:10.2527/jas.2016-0271

39. Li Y, Ning L, Yin Y, Wang R, Zhang Z, Hao L, et al. Age-related shifts in gut microbiota contribute to cognitive decline in aged rats. Aging (Albany NY). 2020;12: 7801–7817. doi:10.18632/aging.103093

40. Mehanna OM, El Askary A, Al-Shehri S, El-Esawy B. Effect of phosphodiesterase inhibitors on renal functions and oxidant/antioxidant parameters in streptozocin-induced diabetic rats. Archives of Physiology and Biochemistry. 2018;124: 424–429. doi:10.1080/13813455.2017.1419267

41. Saipin N, Semsirmboon S, Rungsiwiwut R, Thammacharoen S. High ambient temperature directly decreases milk synthesis in the mammary gland in Saanen goats. Journal of Thermal Biology. 2020;94: 102783. doi:10.1016/j.jtherbio.2020.102783

42. Tomas-Sanchez C, Blanco-Alvarez V-M, Martinez-Fong D, Gonzalez-Barrios J-A, Gonzalez-Vazquez A, Aguilar-Peralta A-K, et al. Prophylactic Zinc and Therapeutic Selenium Administration Increases the Antioxidant Enzyme Activity in the Rat Temporoparietal Cortex and Improves Memory after a Transient Hypoxia-Ischemia. Oxid Med Cell Longev. 2018;2018: 9416432. doi:10.1155/2018/9416432

43. Tabatabaei A, Babaee M, Moradi N, Nabavi M, Arshi S, Fallah S. Serum Concentration of Selenium and GPX Enzyme Activity in Iranian Children with Asthma. Mod Care J. 2020;17. doi:10.5812/modernc102396

44. Pesti-Asbóth G, Molnár-Bíróné P, Forgács I, Remenyik J, Dobránszki J. Ultrasonication affects the melatonin and auxin levels and the antioxidant system in potato in vitro. Front Plant Sci. 2022;13: 979141. doi:10.3389/fpls.2022.979141

45. Szabó K, Gesztelyi R, Lampé N, Kiss R, Remenyik J, Pesti-Asbóth G, et al. Fenugreek (Trigonella Foenum-Graecum) Seed Flour and Diosgenin Preserve Endothelium-Dependent Arterial Relaxation in a Rat Model of Early-Stage Metabolic Syndrome. Int J Mol Sci. 2018;19: E798. doi:10.3390/ijms19030798

46. Villa-Bellosta R. Dietary magnesium supplementation improves lifespan in a mouse model of progeria. EMBO Mol Med. 2020;12: e12423. doi:10.15252/emmm.202012423

47. Lozano I, Van der Werf R, Bietiger W, Seyfritz E, Peronet C, Pinget M, et al. High-fructose and high-fat diet-induced disorders in rats: impact on diabetes risk, hepatic and vascular complications. Nutr Metab (Lond). 2016;13: 15. doi:10.1186/s12986-016-0074-1

48. Almatroodi SA, Alnuqaydan AM, Babiker AY, Almogbel MA, Khan AA, Husain Rahmani A. 6-Gingerol, a Bioactive Compound of Ginger Attenuates Renal Damage in Streptozotocin-Induced Diabetic Rats by Regulating the Oxidative Stress and Inflammation. Pharmaceutics. 2021;13: 317. doi:10.3390/pharmaceutics13030317

49. Park J, Kwon OS, Cho SY, Paick J-S, Kim SW. Chronic administration of atorvastatin could partially ameliorate erectile function in streptozotocin-induced diabetic rats. PLoS One. 2017;12: e0172751. doi:10.1371/journal.pone.0172751

50. Rah DK, Min HJ, Kim YW, Cheon YW. Effect of Platelet-Rich Plasma on Ischemia-Reperfusion Injury in a Skin Flap Mouse Model. Int J Med Sci. 2017;14: 829–839. doi:10.7150/ijms.19573

51. Ellman GL. Tissue sulfhydryl groups. Archives of Biochemistry and Biophysics. 1959;82: 70–77. doi:10.1016/0003-9861(59)90090-6

52. da Rosa G, Alba DF, Silva AD, Gris A, Mendes RE, Mostardeiro VB, et al. Impact of Escherichia coli infection in broiler breeder chicks: The effect of oxidative stress on weight gain. Microbial Pathogenesis. 2020;139: 103861. doi:10.1016/j.micpath.2019.103861

53. Fellenberg M, Delporte C, Backhouse N, Peña I, Speisky H. Effect of dried extract of boldo (Peumus Boldus Mol.) on growth and oxidative tissue status of broiler chickens. Rev Bras Cienc Avic. 2008;10: 245–252. doi:10.1590/S1516-635X2008000400009

54. Hu M-L. [41] Measurement of protein thiol groups and glutathione in plasma. Methods in Enzymology. Elsevier; 1994. pp. 380–385. doi:10.1016/S0076-6879(94)33044-1

55. Doumas BT, Ard Watson W, Biggs HG. Albumin standards and the measurement of serum albumin with bromcresol green. Clinica Chimica Acta. 1971;31: 87–96. doi:10.1016/0009-8981(71)90365-2

56. Fu X, Cate SA, Dominguez M, Osborn W, Özpolat T, Konkle BA, et al. Cysteine Disulfides (Cys-ss-X) as Sensitive Plasma Biomarkers of Oxidative Stress. Scientific Reports. 2019;9: 115. doi:10.1038/s41598-018-35566-2

57. Gyongyosi A, Docs O, Czimmerer Z, Orosz L, Horvath A, Török O, et al. Measuring Expression Levels of Small Regulatory RNA Molecules from Body Fluids and Formalin-Fixed, Paraffin-Embedded Samples. In: Alvarez ML, Nourbakhsh M, editors. RNA Mapping: Methods and Protocols. New York, NY: Springer; 2014. pp. 105–119. doi:10.1007/978-1-4939-1062-5_10

58. Fanatico AC, Pillai PB, Hester PY, Falcone C, Mench JA, Owens CM, et al. Performance, Livability, and Carcass Yield of Slow- and Fast-Growing Chicken Genotypes Fed Low-Nutrient or Standard Diets and Raised Indoors or with Outdoor Access. Poultry Science. 2008;87: 1012–1021. doi:10.3382/ps.2006-00424

59. Cygan-Szczegielniak D, Bogucka J. Growth Performance, Carcass Characteristics and Meat Quality of Organically Reared Broiler Chickens Depending on Sex. Animals. 2021;11: 3274. doi:10.3390/ani11113274

60. Canoğulları S, Baylan M, Bulancak A, Ayaşan T. Differences in performance, carcass characteristics and meat quality between fast- and slow-growing broiler genotypes. 2019 [cited 1 Mar 2023]. doi:10.23751/pn.v21i3.7747

61. Tudorache M, Custura I, Gheorghe A, Habeanu M, Lefter NA, Pogurschi EN, et al. Effects of Genotype and Diet on Performance, Carcass Traits, and Blood Profiles of Slow-Growing Chicks Obtained by Crosses of Local Breed with Commercial Genotype. Agriculture. 2022;12: 1906. doi:10.3390/agriculture12111906

62. Checa J, Aran JM. Reactive Oxygen Species: Drivers of Physiological and Pathological Processes. JIR. 2020;Volume 13: 1057–1073. doi:10.2147/JIR.S275595

63. Birk J, Meyer M, Aller I, Hansen HG, Odermatt A, Dick TP, et al. Endoplasmic reticulum: reduced and oxidized glutathione revisited. J Cell Sci. 2013;126: 1604–1617. doi:10.1242/jcs.117218

64. Rahman K. Studies on free radicals, antioxidants, and co-factors. Clin Interv Aging. 2007;2: 219–236.

65. Babinszky L, Horváth M, Remenyik J, Verstegen MWA. The adverse effects of heat stress on the antioxidant status and performance of pigs and poultry and reducing these effects with nutritional tools. Poultry and pig nutrition: Challenges of the 21st century. 2019; 187–208. doi:10.3920/978-90-8686-884-1_8

66. Ayala A, Muñoz MF, Argüelles S. Lipid Peroxidation: Production, Metabolism, and Signaling Mechanisms of Malondialdehyde and 4-Hydroxy-2-Nonenal. Oxid Med Cell Longev. 2014;2014: 360438. doi:10.1155/2014/360438

67. Pizzino G, Irrera N, Cucinotta M, Pallio G, Mannino F, Arcoraci V, et al. Oxidative Stress: Harms and Benefits for Human Health. Oxid Med Cell Longev. 2017;2017: 8416763. doi:10.1155/2017/8416763

68. Kurutas EB. The importance of antioxidants which play the role in cellular response against oxidative/nitrosative stress: current state. Nutr J. 2016;15: 71. doi:10.1186/s12937-016-0186-5

69. Szarka A, Tomasskovics B, Bánhegyi G. The Ascorbate-glutathione-α-tocopherol Triad in Abiotic Stress Response. Int J Mol Sci. 2012;13: 4458–4483. doi:10.3390/ijms13044458

70. Miazek K, Beton K, Śliwińska A, Brożek-Płuska B. The Effect of β-Carotene, Tocopherols and Ascorbic Acid as Anti-Oxidant Molecules on Human and Animal In Vitro/In Vivo Studies: A Review of Research Design and Analytical Techniques Used. Biomolecules. 2022;12: 1087. doi:10.3390/biom12081087

71. Barouh N, Bourlieu-Lacanal C, Figueroa-Espinoza MC, Durand E, Villeneuve P. Tocopherols as antioxidants in lipid-based systems: The combination of chemical and physicochemical interactions determines their efficiency. Comp Rev Food Sci Food Safe. 2022;21: 642–688. doi:10.1111/1541-4337.12867

72. Kumar A, Prasad A, Pospíšil P. Formation of α-tocopherol hydroperoxide and α-tocopheroxyl radical: relevance for photooxidative stress in Arabidopsis. Sci Rep. 2020;10: 19646. doi:10.1038/s41598-020-75634-0

73. Jiang Q. Natural forms of vitamin E: metabolism, antioxidant and anti-inflammatory activities and the role in disease prevention and therapy. Free Radic Biol Med. 2014;72: 76–90. doi:10.1016/j.freeradbiomed.2014.03.035

74. Martemucci G, Costagliola C, Mariano M, D'andrea L, Napolitano P, D'Alessandro AG. Free Radical Properties, Source and Targets, Antioxidant Consumption and Health. Oxygen. 2022;2: 48–78. doi:10.3390/oxygen2020006

75. Aquilano K, Baldelli S, Ciriolo MR. Glutathione: new roles in redox signaling for an old antioxidant. Frontiers in Pharmacology. 2014;5. Available: https://www.frontiersin.org/articles/10.3389/fphar.2014.00196

76. Noctor G, Queval G, Mhamdi A, Chaouch S, Foyer CH. Glutathione. Arabidopsis Book. 2011;9: e0142. doi:10.1199/tab.0142

77. Weaver K, Skouta R. The Selenoprotein Glutathione Peroxidase 4: From Molecular Mechanisms to Novel Therapeutic Opportunities. Biomedicines. 2022;10: 891. doi:10.3390/biomedicines10040891

78. Giustarini D, Tsikas D, Colombo G, Milzani A, Dalle-Donne I, Fanti P, et al. Pitfalls in the analysis of the physiological antioxidant glutathione (GSH) and its disulfide (GSSG) in biological samples: An elephant in the room. J Chromatogr B Analyt Technol Biomed Life Sci. 2016;1019: 21–28. doi:10.1016/j.jchromb.2016.02.015

79. Pizzorno J. Glutathione! Integr Med (Encinitas). 2014;13: 8–12.

80. El-Senousey HK, Chen B, Wang JY, Atta AM, Mohamed FR, Nie QH. Effects of dietary vitamin C, vitamin E, and alpha-lipoic acid supplementation on the antioxidant defense system and immune-related gene expression in broilers exposed to oxidative stress by dexamethasone. Poultry Science. 2018;97: 30–38. doi:10.3382/ps/pex298

81. Musalmah M, Fairuz AH, Gapor MT, Ngah WZW. Effect of vitamin E on plasma malondialdehyde, antioxidant enzyme levels and the rates of wound closures during wound healing in normal and diabetic rats. Asia Pac J Clin Nutr. 2002;11 Suppl 7: S448-451. doi:10.1046/j.1440-6047.11.s.7.6.x

82. Khare M, Mohanty C, Das BK, Jyoti A, Mukhopadhyay B, Mishra SP. Free Radicals and Antioxidant Status in Protein Energy Malnutrition. International Journal of Pediatrics. 2014;2014: e254396. doi:10.1155/2014/254396

83. Tóthová C, Sesztáková E, Bielik B, Nagy O. Changes of total protein and protein fractions in broiler chickens during the fattening period. Veterinary World. 2019;12: 598. doi:10.14202/vetworld.2019.598-604

84. Rezende MS, Mundim AV, Fonseca BB, Miranda RL, Oliveira W, Lellis CG. Profile of Serum Metabolites and Proteins of Broiler Breeders in Rearing Age. Braz J Poult Sci. 2017;19: 583–586. doi:10.1590/1806-9061-2016-0338

85. Mondal D, Chattopadhyay S, Batabyal S, Bera A, Bhattacharya D. Plasma biochemical indices at various stages of infection with a field isolate of Eimeria tenella in broiler chicken. Vet World. 2011; 404. doi:10.5455/vetworld.2011.404-409

86. Soeters PB, Wolfe RR, Shenkin A. Hypoalbuminemia: Pathogenesis and Clinical Significance. JPEN J Parenter Enteral Nutr. 2019;43: 181–193. doi:10.1002/jpen.1451

87. Taverna M, Marie A-L, Mira J-P, Guidet B. Specific antioxidant properties of human serum albumin. Annals of Intensive Care. 2013;3: 4. doi:10.1186/2110-5820-3-4

88. Roche M, Rondeau P, Singh NR, Tarnus E, Bourdon E. The antioxidant properties of serum albumin. FEBS Letters. 2008;582: 1783–1787. doi:10.1016/j.febslet.2008.04.057

89. Medina-Navarro R, Durán-Reyes G, Díaz-Flores M, Vilar-Rojas C. Protein Antioxidant Response to the Stress and the Relationship between Molecular Structure and Antioxidant Function. PLOS ONE. 2010;5: e8971. doi:10.1371/journal.pone.0008971

90. B H. Albumin--an important extracellular antioxidant? Biochemical pharmacology. 1988;37. doi:10.1016/0006-2952(88)90126-8

91. Z. Penezić A, B. Jovanović V, D. Pavićević I, M. Aćimović J, M. Mandić L. HSA carbonylation with methylglyoxal and the binding/release of copper(ii) ions. Metallomics. 2015;7: 1431–1438. doi:10.1039/C5MT00159E

92. Khosravifarsani M, Monfared AS, Pouramir M, Zabihi E. Effects of Fenton Reaction on Human Serum Albumin: An In Vitro Study. Electron Physician. 2016;8: 2970–2976. doi:10.19082/2970

93. Nakashima F, Shibata T, Kamiya K, Yoshitake J, Kikuchi R, Matsushita T, et al. Structural and functional insights into S-thiolation of human serum albumins. Sci Rep. 2018;8: 932. doi:10.1038/s41598-018-19610-9

94. Taverna M, Marie A-L, Mira J-P, Guidet B. Specific antioxidant properties of human serum albumin. Ann Intensive Care. 2013;3: 4. doi:10.1186/2110-5820-3-4

95. Anthony-Regnitz CM, Wilson AE, Sweazea KL, Braun EJ. Fewer Exposed Lysine Residues May Explain Relative Resistance of Chicken Serum Albumin to In Vitro Protein Glycation in Comparison to Bovine Serum Albumin. J Mol Evol. 2020;88: 653–661. doi:10.1007/s00239-020-09964-y

96. Belinskaia DA, Voronina PA, Shmurak VI, Jenkins RO, Goncharov NV. Serum Albumin in Health and Disease: Esterase, Antioxidant, Transporting and Signaling Properties. Int J Mol Sci. 2021;22: 10318. doi:10.3390/ijms221910318

97. Braidy N, Zarka M, Jugder B-E, Welch J, Jayasena T, Chan DKY, et al. The Precursor to Glutathione (GSH), γ-Glutamylcysteine (GGC), Can Ameliorate Oxidative Damage and Neuroinflammation Induced by Aβ40 Oligomers in Human Astrocytes. Frontiers in Aging Neuroscience. 2019;11. Available: https://www.frontiersin.org/articles/10.3389/fnagi.2019.00177

98. Nte I, Gunn H. Cysteine in Broiler Poultry Nutrition. 2021. doi:10.5772/intechopen.97281

99. Werge MP, McCann A, Galsgaard ED, Holst D, Bugge A, Albrechtsen NJW, et al. The Role of the Transsulfuration Pathway in Non-Alcoholic Fatty Liver Disease. Journal of Clinical Medicine. 2021;10: 1081. doi:10.3390/jcm10051081

100. Garrido Ruiz D, Sandoval-Perez A, Rangarajan AV, Gunderson EL, Jacobson MP. Cysteine Oxidation in Proteins: Structure, Biophysics, and Simulation. Biochemistry. 2022;61: 2165–2176. doi:10.1021/acs.biochem.2c00349

101. Turell L, Radi R, Alvarez B. The thiol pool in human plasma: The central contribution of albumin to redox processes. Free Radical Biology and Medicine. 2013;65: 244–253. doi:10.1016/j.freeradbiomed.2013.05.050

102. Phaniendra A, Jestadi DB, Periyasamy L. Free Radicals: Properties, Sources, Targets, and Their Implication in Various Diseases. Indian J Clin Biochem. 2015;30: 11–26. doi:10.1007/s12291-014-0446-0

103. Aquilano K, Baldelli S, Ciriolo MR. Glutathione: new roles in redox signaling for an old antioxidant. Frontiers in Pharmacology. 2014;5. Available: https://www.frontiersin.org/articles/10.3389/fphar.2014.00196

104. Glutathione: Role in Oxidative/Nitrosative Stress, Antioxidant Defense, and Treatments - Raj Rai - 2021 - ChemistrySelect - Wiley Online Library. [cited 3 Jan 2023]. Available: https://chemistry-europe.onlinelibrary.wiley.com/doi/10.1002/slct.202100773

105. Sibrian-Vazquez M, Escobedo JO, Lim S, Samoei GK, Strongin RM. Homocystamides promote free-radical and oxidative damage to proteins. Proc Natl Acad Sci USA. 2010;107: 551–554. doi:10.1073/pnas.0909737107

106. Altomare A, Baron G, Brioschi M, Longoni M, Butti R, Valvassori E, et al. N-Acetyl-Cysteine Regenerates Albumin Cys34 by a Thiol-Disulfide Breaking Mechanism: An Explanation of Its Extracellular Antioxidant Activity. Antioxidants (Basel). 2020;9: 367. doi:10.3390/antiox9050367

107. Aldini G, Altomare A, Baron G, Vistoli G, Carini M, Borsani L, et al. N-Acetylcysteine as an antioxidant and disulphide breaking agent: the reasons why. Free Radical Research. 2018;52: 751–762. doi:10.1080/10715762.2018.1468564

108. Fu X, Cate S, Dominguez M, Osborn W, Ozpolat T, Konkle B, et al. Cysteine Disulfides (Cys-ss-X) as Sensitive Plasma Biomarkers of Oxidative Stress. Scientific Reports. 2019;9. doi:10.1038/s41598-018-35566-2

109. Surai P. Antioxidant Systems in Poultry Biology: Superoxide Dismutase. Animal Nutrition. 2016;1: 8. doi:10.21767/2572-5459.100008

110. Sbodio JI, Snyder SH, Paul BD. Regulators of the transsulfuration pathway. British Journal of Pharmacology. 2019;176: 583–593. doi:10.1111/bph.14446

111. Baudouin-Cornu P, Lagniel G, Kumar C, Huang M-E, Labarre J. Glutathione Degradation Is a Key Determinant of Glutathione Homeostasis. J Biol Chem. 2012;287: 4552–4561. doi:10.1074/jbc.M111.315705

112. Jones DP, Carlson JL, Mody VC, Cai J, Lynn MJ, Sternberg P. Redox state of glutathione in human plasma. Free Radical Biology and Medicine. 2000;28: 625–635. doi:10.1016/S0891-5849(99)00275-0

113. Lu SC. REGULATION OF GLUTATHIONE SYNTHESIS. Mol Aspects Med. 2009;30: 42–59. doi:10.1016/j.mam.2008.05.005

114. The Role of Thiols and Disulfides on Protein Stability: Ingenta Connect. [cited 4 Jan 2023]. Available: https://www.ingentaconnect.com/content/ben/cpps/2009/00000010/00000006/art00008

115. Poole LB. The basics of thiols and cysteines in redox biology and chemistry. Free Radical Biology and Medicine. 2015;80: 148–157. doi:10.1016/j.freeradbiomed.2014.11.013

116. Requejo R, Hurd TR, Costa NJ, Murphy MP. Cysteine residues exposed on protein surfaces are the dominant intramitochondrial thiol and may protect against oxidative damage. FEBS J. 2010;277: 1465–1480. doi:10.1111/j.1742-4658.2010.07576.x

117. Hansen RE, Roth D, Winther JR. Quantifying the global cellular thiol-disulfide status. Proc Natl Acad Sci U S A. 2009;106: 422–427. doi:10.1073/pnas.0812149106

118. Turell L, Radi R, Alvarez B. The thiol pool in human plasma: The central contribution of albumin to redox processes. Free Radic Biol Med. 2013;65: 244–253. doi:10.1016/j.freeradbiomed.2013.05.050

119. Werge MP, McCann A, Galsgaard ED, Holst D, Bugge A, Albrechtsen NJW, et al. The Role of the Transsulfuration Pathway in Non-Alcoholic Fatty Liver Disease. Journal of Clinical Medicine. 2021;10: 1081. doi:10.3390/jcm10051081

120. Ramos-Tovar E, Muriel P. Molecular Mechanisms That Link Oxidative Stress, Inflammation, and Fibrosis in the Liver. Antioxidants (Basel). 2020;9: 1279. doi:10.3390/antiox9121279

121. Tilg H. The Role of Cytokines in Non-Alcoholic Fatty Liver Disease. DDI. 2010;28: 179–185. doi:10.1159/000282083

122. Nallagangula KS, Nagaraj SK, Venkataswamy L, Chandrappa M. Liver fibrosis: a compilation on the biomarkers status and their significance during disease progression. Future Sci OA. 2017;4: FSO250. doi:10.4155/fsoa-2017-0083

123. Smirne C, Croce E, Di Benedetto D, Cantaluppi V, Comi C, Sainaghi PP, et al. Oxidative Stress in Non-Alcoholic Fatty Liver Disease. Livers. 2022;2: 30–76. doi:10.3390/livers2010003

124. Luedde T, Schwabe RF. NF-κB in the liver--linking injury, fibrosis and hepatocellular carcinoma. Nat Rev Gastroenterol Hepatol. 2011;8: 108–118. doi:10.1038/nrgastro.2010.213

125. Sen N, Paul BD, Gadalla MM, Mustafa AK, Sen T, Xu R, et al. Hydrogen sulfide-linked sulfhydration of NF-κB mediates its anti-apoptotic actions. Mol Cell. 2012;45: 13–24. doi:10.1016/j.molcel.2011.10.021

126. Kogut MH. Cytokines and prevention of infectious diseases in poultry: A review. Avian Pathology. 2000;29: 395–404. doi:10.1080/030794500750047135

127. Jeurissen SHM, Boonstra-Blom AG, Al-Garib SO, Hartog L, Koch G. Defence mechanisms against viral infection in poultry: A review. Veterinary Quarterly. 2000;22: 204–208. doi:10.1080/01652176.2000.9695059

128. Vazquez MI, Catalan-Dibene J, Zlotnik A. B cells responses and cytokine production are regulated by their immune microenvironment. Cytokine. 2015;74: 318–326. doi:10.1016/j.cyto.2015.02.007

129. Oeckinghaus A, Ghosh S. The NF-κB Family of Transcription Factors and Its Regulation. Cold Spring Harb Perspect Biol. 2009;1: a000034. doi:10.1101/cshperspect.a000034

130. Liu T, Zhang L, Joo D, Sun S-C. NF-κB signaling in inflammation. Signal Transduct Target Ther. 2017;2: 17023. doi:10.1038/sigtrans.2017.23

131. Surai PF, Kochish II, Kidd MT. Redox Homeostasis in Poultry: Regulatory Roles of NF-κB. Antioxidants (Basel). 2021;10: 186. doi:10.3390/antiox10020186

132. Go Y-M, Jones DP. Intracellular Proatherogenic Events and Cell Adhesion Modulated by Extracellular Thiol/Disulfide Redox State. Circulation. 2005;111: 2973–2980. doi:10.1161/CIRCULATIONAHA.104.515155

133. Iyer SS, Accardi CJ, Ziegler TR, Blanco RA, Ritzenthaler JD, Rojas M, et al. Cysteine Redox Potential Determines Pro-Inflammatory IL-1β Levels. PLOS ONE. 2009;4: e5017. doi:10.1371/journal.pone.0005017

134. Biswas SK. Does the Interdependence between Oxidative Stress and Inflammation Explain the Antioxidant Paradox? Oxidative Medicine and Cellular Longevity. 2016;2016: 1–9. doi:10.1155/2016/5698931

135. Serviddio G, Bellanti F, Vendemiale G. Free radical biology for medicine: learning from nonalcoholic fatty liver disease. Free Radical Biology and Medicine. 2013;65: 952–968. doi:10.1016/j.freeradbiomed.2013.08.174

136. Kanda T, Matsuoka S, Yamazaki M, Shibata T, Nirei K, Takahashi H, et al. Apoptosis and non-alcoholic fatty liver diseases. World J Gastroenterol. 2018;24: 2661–2672. doi:10.3748/wjg.v24.i25.2661

137. Delli Bovi AP, Marciano F, Mandato C, Siano MA, Savoia M, Vajro P. Oxidative Stress in Non-alcoholic Fatty Liver Disease. An Updated Mini Review. Front Med (Lausanne). 2021;8: 595371. doi:10.3389/fmed.2021.595371

138. Li S, Hong M, Tan H-Y, Wang N, Feng Y. Insights into the Role and Interdependence of Oxidative Stress and Inflammation in Liver Diseases. Oxidative Medicine and Cellular Longevity. 2016;2016: e4234061. doi:10.1155/2016/4234061

139. Heeba GH, Mahmoud ME. Therapeutic potential of morin against liver fibrosis in rats: modulation of oxidative stress, cytokine production and nuclear factor kappa B. Environ Toxicol Pharmacol. 2014;37: 662–671. doi:10.1016/j.etap.2014.01.026

140. Ganz M, Bukong TN, Csak T, Saha B, Park J-K, Ambade A, et al. Progression of non-alcoholic steatosis to steatohepatitis and fibrosis parallels cumulative accumulation of danger signals that promote inflammation and liver tumors in a high fat-cholesterol-sugar diet model in mice. J Transl Med. 2015;13: 193. doi:10.1186/s12967-015-0552-7

141. Czaja AJ. Hepatic inflammation and progressive liver fibrosis in chronic liver disease. World J Gastroenterol. 2014;20: 2515–2532. doi:10.3748/wjg.v20.i10.2515

142. Mani S, Li H, Yang G, Wu L, Wang R. Deficiency of cystathionine gamma-lyase and hepatic cholesterol accumulation during mouse fatty liver development. Science Bulletin. 2015;60: 336–347. doi:10.1007/s11434-014-0722-7

143. Watanabe M, Osada J, Aratani Y, Kluckman K, Reddick R, Malinow MR, et al. Mice deficient in cystathionine beta-synthase: animal models for mild and severe homocyst(e)inemia. Proceedings of the National Academy of Sciences. 1995;92: 1585–1589. doi:10.1073/pnas.92.5.1585

144. Robert K, Nehmé J, Bourdon E, Pivert G, Friguet B, Delcayre C, et al. Cystathionine β Synthase Deficiency Promotes Oxidative Stress, Fibrosis, and Steatosis in Mice Liver. Gastroenterology. 2005;128: 1405–1415. doi:10.1053/j.gastro.2005.02.034

145. Hamelet J, Demuth K, Paul J-L, Delabar J-M, Janel N. Hyperhomocysteinemia due to cystathionine beta synthase deficiency induces dysregulation of genes involved in hepatic lipid homeostasis in mice. Journal of Hepatology. 2007;46: 151–159. doi:10.1016/j.jhep.2006.07.028

146. Guillén N, Navarro MA, Arnal C, Noone E, Arbonés-Mainar JM, Acín S, et al. Microarray analysis of hepatic gene expression identifies new genes involved in steatotic liver. Physiological Genomics. 2009;37: 187–198. doi:10.1152/physiolgenomics.90339.2008

147. Stipanuk MH, Ueki I. Dealing with methionine/homocysteine sulfur: cysteine metabolism to taurine and inorganic sulfur. Journal of Inherited Metabolic Disease. 2011;34: 17–32. doi:10.1007/s10545-009-9006-9

148. Chernigova SV, Chernigov YV, Vatnikov YA, Kulikov EV, Popova IA, Shirmanov VI, et al. Special aspects of systemic inflammation course in animals. Vet World. 2019;12: 932–937. doi:10.14202/vetworld.2019.932-937

149. Sagristá ML, GarcÍa AF, De Madariaga MA, Mora M. Antioxidant and Pro-oxidant Effect of the Thiolic Compounds N -acetyl- l -cysteine and Glutathione against Free Radical-induced Lipid Peroxidation. Free Radical Research. 2002;36: 329–340. doi:10.1080/10715760290019354

150. Domínguez R, Pateiro M, Gagaoua M, Barba FJ, Zhang W, Lorenzo JM. A Comprehensive Review on Lipid Oxidation in Meat and Meat Products. Antioxidants. 2019;8: 429. doi:10.3390/antiox8100429

151. Pehlivan FE. Vitamin C: An Antioxidant Agent. Vitamin C. IntechOpen; 2017. doi:10.5772/intechopen.69660

152. Belinskaia DA, Voronina PA, Shmurak VI, Jenkins RO, Goncharov NV. Serum Albumin in Health and Disease: Esterase, Antioxidant, Transporting and Signaling Properties. Int J Mol Sci. 2021;22: 10318. doi:10.3390/ijms221910318

153. Xing T, Pan X, Zhang L, Gao F. Hepatic Oxidative Stress, Apoptosis, and Inflammation in Broiler Chickens With Wooden Breast Myopathy. Front Physiol. 2021;12: 659777. doi:10.3389/fphys.2021.659777

154. Jensen K, Gluud C. The mallory body: Morphological, clinical and experimental studies (part 1 of a literature survey). Hepatology. 1994;20: 1061–1077. doi:10.1002/hep.1840200440

155. Jensen K, Gluud C. The mallory body: Theories on development and pathological significance (part 2 of a literature survey). Hepatology. 1994;20: 1330–1342. doi:10.1002/hep.1840200534

Figure

Click here to access/download;Figure;Fig 1.tif

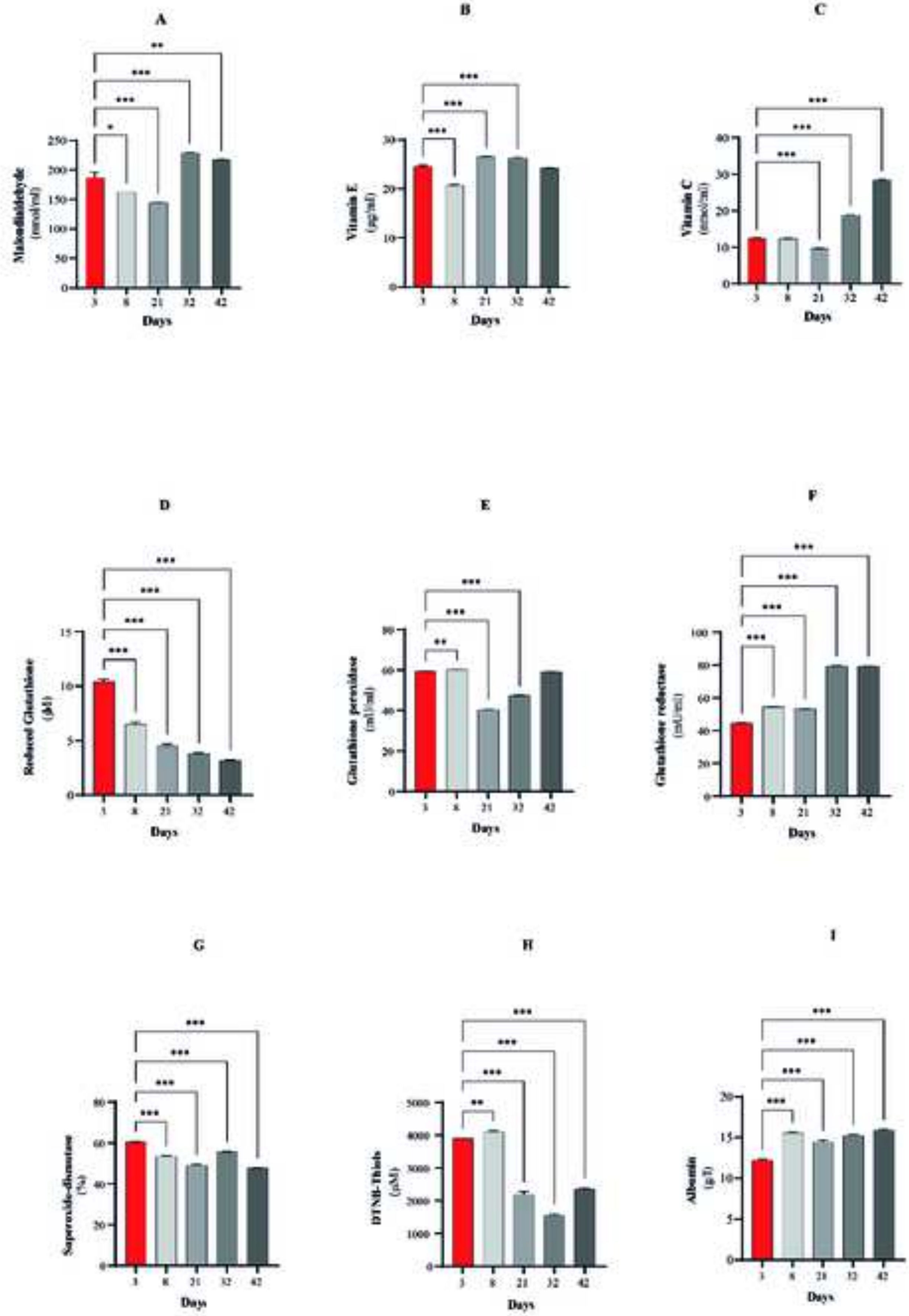

Figure

Click here to access/download;Figure;Fig 2.tif

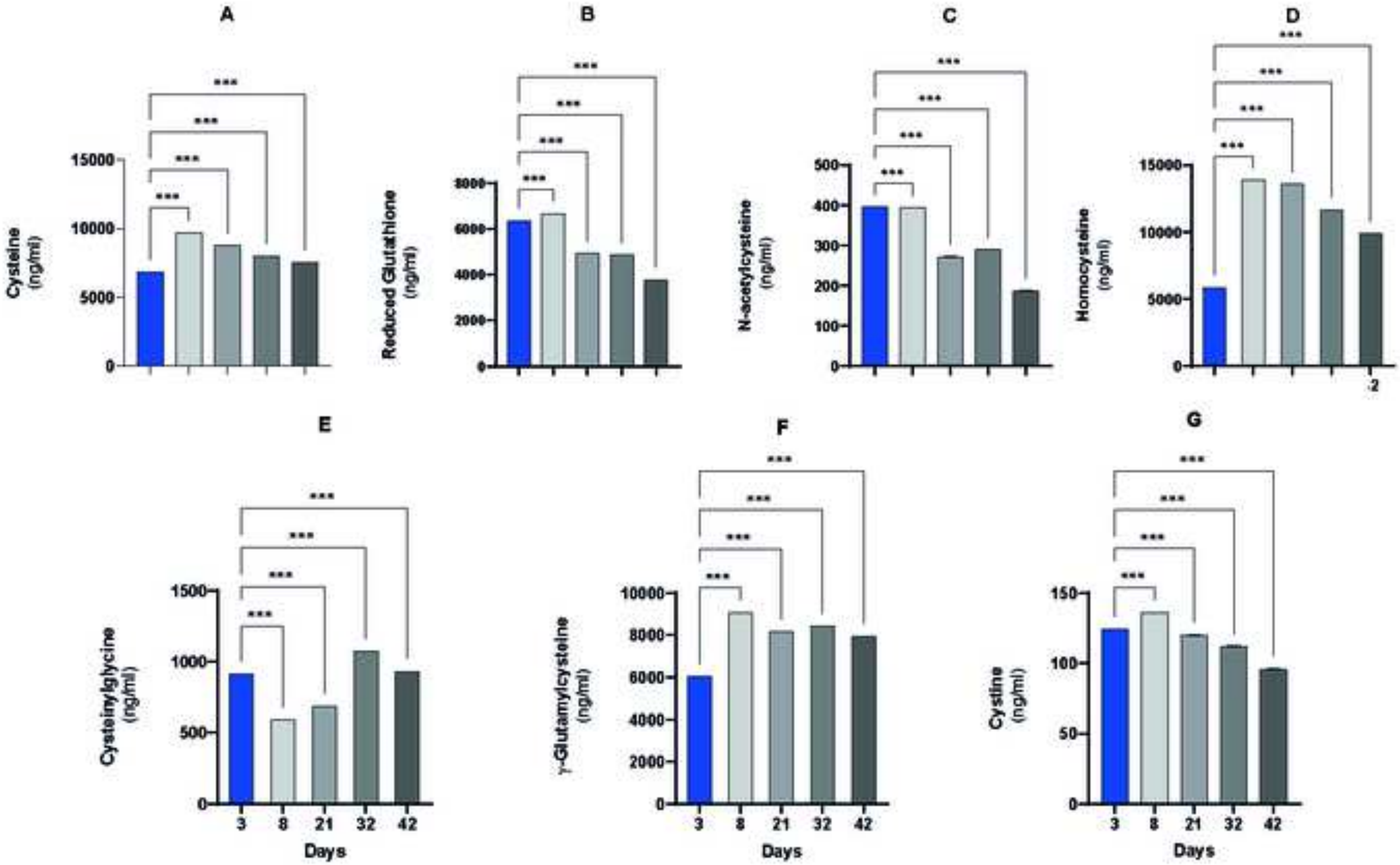

Figure

Click here to access/download;Figure;Fig 3.tif

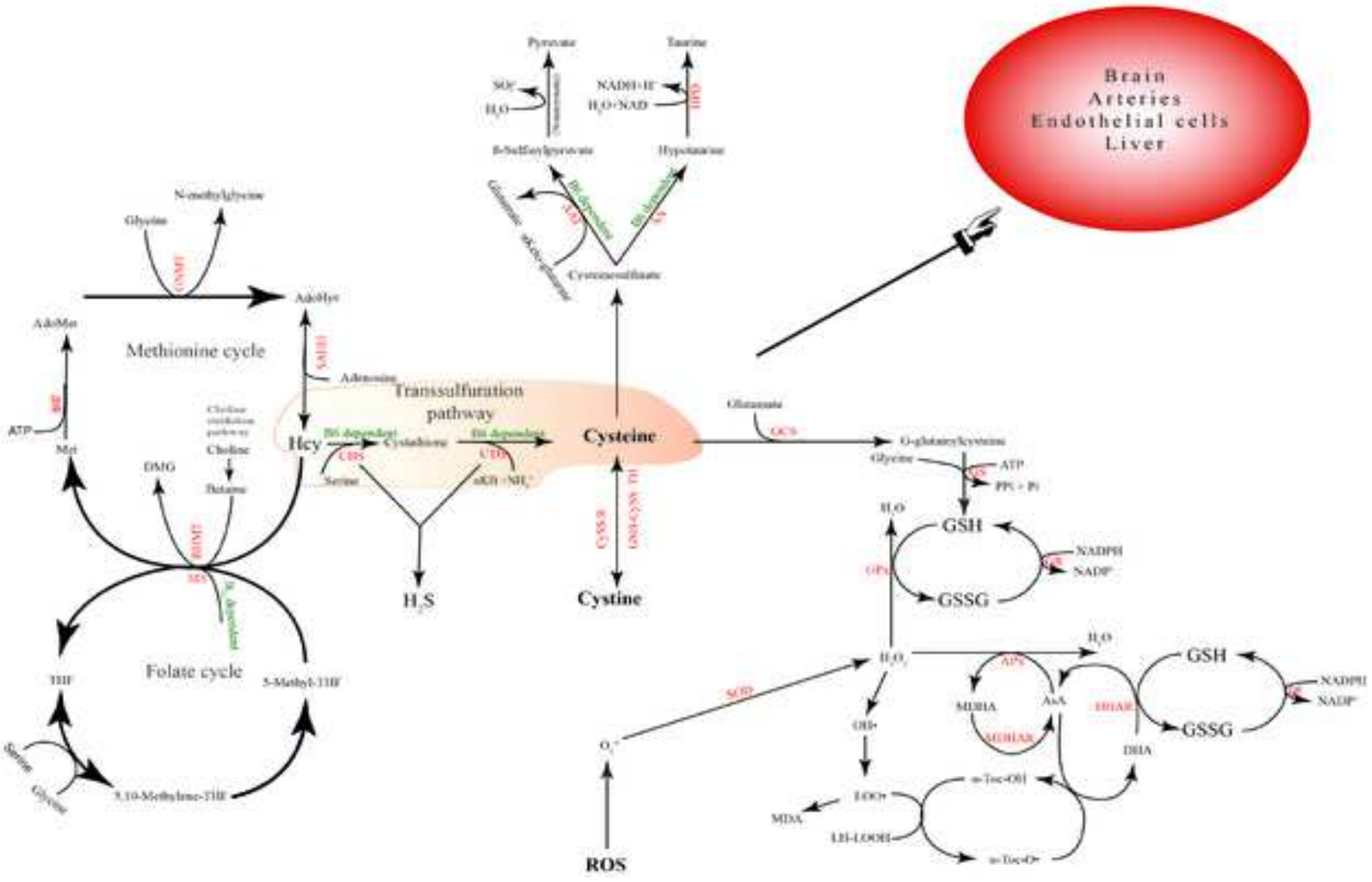

Figure

Click here to access/download;Figure;Fig 4.tif

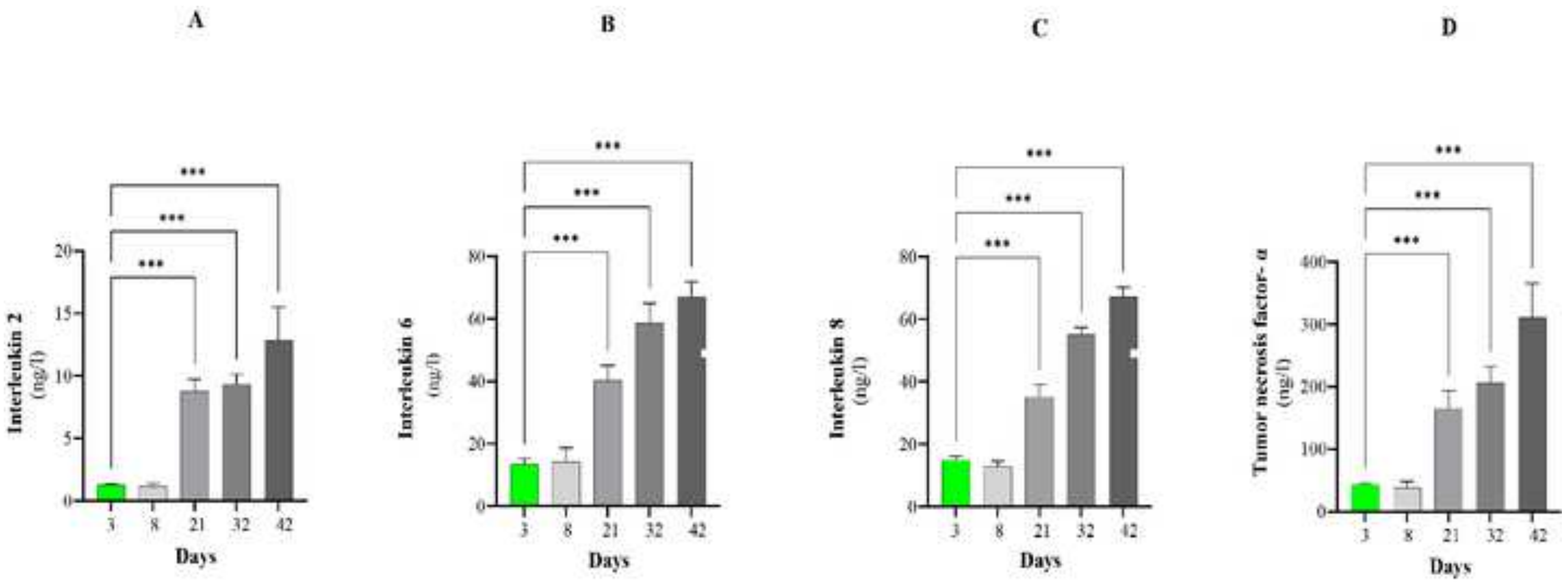

Figure Click here to access/download;Figure;Fig 5.tif

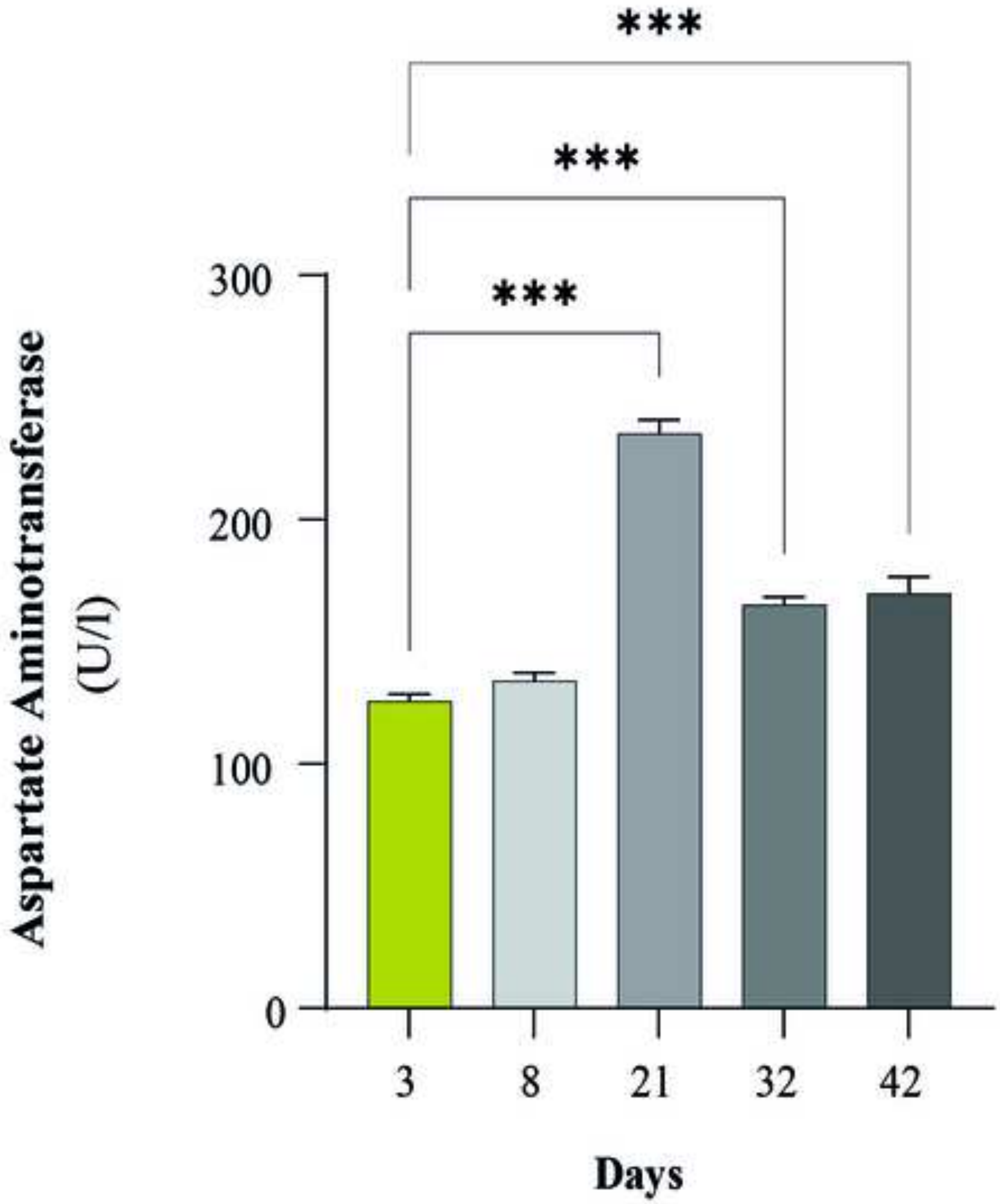

Figure

Click here to access/download;Figure;Fig 6.tif

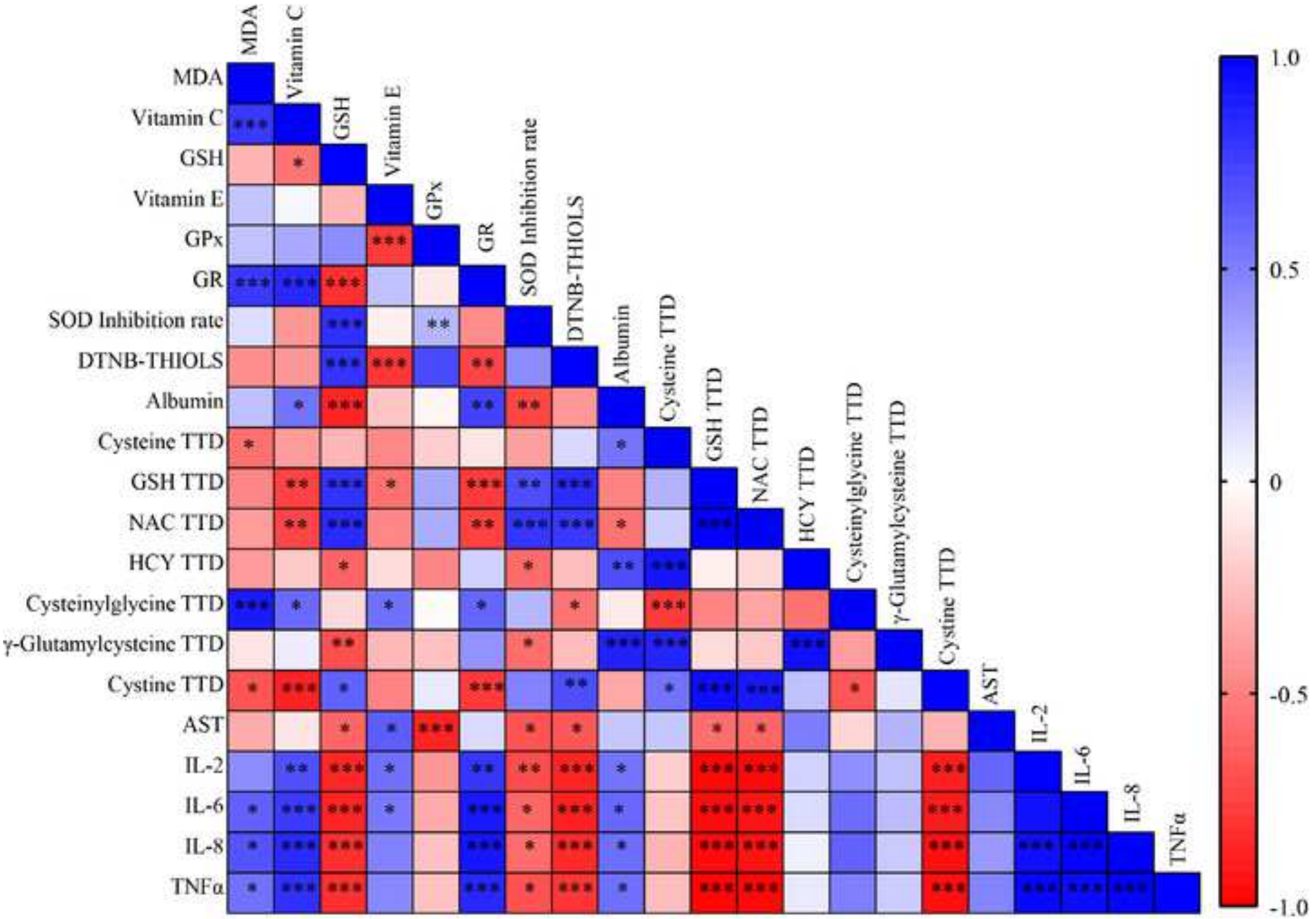

A B

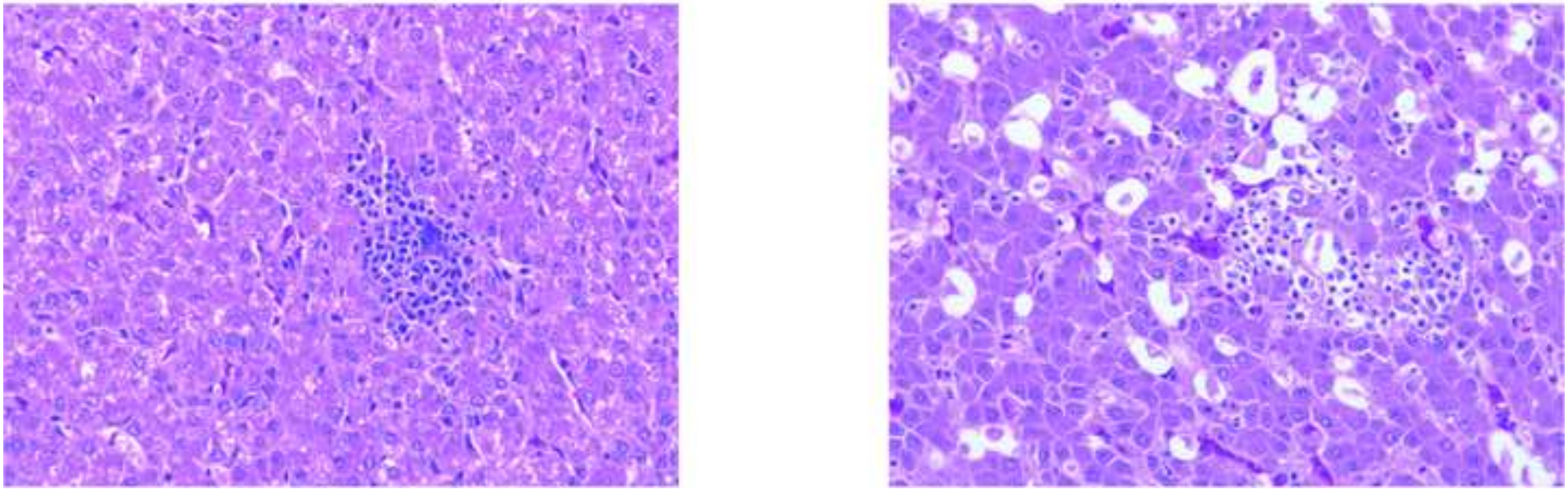

Supporting Information cerificate

Click here to access/download
Supporting Information
ZWJZB1PR_8BBC-8696-11B9-855D-F572.pdf

Click here to access/download
Preprint PDF
Manuscript.pdf
