# Supplementary material for: Monitoring physiological processes of fast-growing broilers during the whole life cycle: Changes of redox-homeostasis effected to trassulfuration pathway predicting the development of non-alcoholic fatty liver disease
Source: PLoS One. 2023 Aug 17;18(8):e0290310. doi: 10.1371/journal.pone.0290310 (PMC10434899; doi:10.1371/journal.pone.0290310)
Supplement: Attachment — Submitted filename: Comments PONE-D-23-15244 (1).docx [file pone.0290310.s002.docx]

Comments/ Suggestions

Manuscript No.: PONE-D-23-15244

Manuscript Title: Monitoring physiological processes of fast-growing broilers during the whole

life cycle: changes of redox-homeostasis effected to trassulfuration pathway

predicting the development of non-alcoholic fatty liver disease

1. Page no. 6, line no. 102: Separate “abovementioned”; it is jumbled.
2. Page no. 6, line no. 114: “Day old” means “first day of chick’s life”. Please avoid to mention “1-day old” from the entire manuscript.
3. Page no. 7, line no. 134: Replace “1” with “single”. Replace “3 birds/pen” with “three birds/ pen”.
4. Page no. 9, line no. 157: Replace “dH_2_O” with “distilled water” and make it uniform for the entire manuscript.
5. Page no. 13, line no. 256: Insert “.” before the word “Both”.
6. Abstract section: “A method was developed to measure the levels of other small molecule thiol components of plasma”.

But in page 12, line 234: The free thiol concentration was determined through Ellman’s assay (Ethos Biosciences Inc., Philadelphia, PA, United States).

Please justify the statement of abstract?

7. Page 13, line 260 – 264: If any new method cited, it should be described by the authors. In case already published method, reference could be cited and a brief account could be presented.
8. Avoid presentation of conclusion in bullet form. It should be presented in sentence case within word count of 150.

**Final decision**: Recommended for accepting the paper with minor correction.
