# Supplementary material for: Monitoring physiological processes of fast-growing broilers during the whole life cycle: Changes of redox-homeostasis effected to trassulfuration pathway predicting the development of non-alcoholic fatty liver disease
Source: PLoS One. 2023 Aug 17;18(8):e0290310. doi: 10.1371/journal.pone.0290310 (PMC10434899; doi:10.1371/journal.pone.0290310)
Supplement: Attachment — Submitted filename: Response to Reviewers.docx [file pone.0290310.s003.docx]

Dear Professor El Basuini,

We have uploaded the revised version of our manuscript entitled “*Monitoring physiological processes of fast-growing broilers during the whole life cycle: changes of redox-homeostasis effected to trassulfuration pathway predicting the development of non-alcoholic fatty liver disease*

*Diagnostic relevance of the redox status in fast-growing broilers during rearing*”

We thank the thorough and detailed analysis, critical comments and suggestions of the reviewers. They were very useful. As it can be seen from the point by point response letter they were accepted, and incorporated. We feel that these improved the scientific level of our manuscript.

We revised the manuscript following the reviewer’s advices and PLOS ONE’s publication criteria.

We hope that with all these changes and corrections the paper is acceptable for PLOS ONE.

Yours sincerely,

Judit Remenyik

***Point by point answers***

Reviewer #1

**“How sample size was determined?”**

The pen size in the experimental barn determined the number of birds to achieve a stocking density used in broiler chicken production (3.9 m2 pens, 60 birds = stocking density of 650 cm^2^/bird)

**“What was experimental design?” and “Which experimental design?”**

Birds were considered as biological replicates for the measured parameters except feed intake, where the three pens provided three data.

**“Mention strain”**

We corrected it. Page no.6, line no.114 “ Ross 308 broiler chickens”.

**“Why too old reference was used when latest Strain Based guidelines are available.”**

Corrected. Page no.6, line no.121.,

In References:

Page no.35., line no. 769-770

30.	Ross-BroilerHandbook2018-EN.pdf. Available: https://aviagen.com/assets/Tech_Center/Ross_Broiler/Ross-BroilerHandbook2018-EN.pdf

**“Add references of each parameter”**

We filled the missing citation.

Page no. 9, line no 168 [33]

In references: page no.35 line no. 778-781

33.	Santolim LV, Amaral MEC do, Fachi JL, Mendes MF, Oliveira CA de. Vitamin E and caloric restriction promote hepatic homeostasis through expression of connexin 26, N-cad, E-cad and cholesterol metabolism genes. The Journal of Nutritional Biochemistry. 2017;39: 86–92. doi:10.1016/j.jnutbio.2016.09.011

Page no. 13, line no. 256. citation [57]

In references: page no.37, line no.855-857

57.	Gane EJ, Weilert F, Orr DW, Keogh GF, Gibson M, Lockhart MM, et al. The mitochondria-targeted anti-oxidant mitoquinone decreases liver damage in a phase II study of hepatitis C patients. Liver International. 2010;30: 1019–1026. doi:10.1111/j.1478-3231.2010.02250.x

Page no. 15, line no.302.; citation [58]

In references: page no. 37, line no.858-860

58.	Fu X, Cate SA, Dominguez M, Osborn W, Özpolat T, Konkle BA, et al. Cysteine Disulfides (Cys-ss-X) as Sensitive Plasma Biomarkers of Oxidative Stress. Scientific Reports. 2019;9: 115. doi:10.1038/s41598-018-35566-2

Page no. 16, line no. 325 citation [59-62]

In reference: page no. 37., line no. 861-875

59.	Wandita TG, Joshi N, Nam IS, Yang SH, Park HS, Hwang SG. Dietary Supplementation of Purified Amino Acid Derived from Animal Blood on Immune Response and Growth Performance of Broiler Chicken. Tropical Animal Science Journal. 2018;41: 108–113. doi:10.5398/tasj.2018.41.2.108

60.	Varasteh S, Braber S, Akbari P, Garssen J, Fink-Gremmels J. Differences in Susceptibility to Heat Stress along the Chicken Intestine and the Protective Effects of Galacto-Oligosaccharides. PLOS ONE. 2015;10: e0138975. doi:10.1371/journal.pone.0138975

61.	Krzysica P, Verhoog L, de Vries S, Smits C, Savelkoul HFJ, Tijhaar E. Optimization of Capture ELISAs for Chicken Cytokines Using Commercially Available Antibodies. Animals. 2022;12: 3040. doi:10.3390/ani12213040

62.	Jarosz ŁS, Marek A, Grądzki Z, Kwiecień M, Kaczmarek B. The effect of feed supplementation with a copper-glycine chelate and copper sulphate on selected humoral and cell-mediated immune parameters, plasma superoxide dismutase activity, ceruloplasmin and cytokine concentration in broiler chickens. J Anim Physiol Anim Nutr. 2018;102: e326–e336. doi:10.1111/jpn.12750

**„add mathematical model for better illustration of data analysis”**

Corrected, page no.16, line no. 331-332.

„The normality assumption was checked by analyzing the ANOVA model residuals using Shapiro-Wilk test. The homogeneity of variance was checked using Levente’s test.”

**„add actual p-value of each result” and„add SEM and actual p-value”**

Corrected, page no. 17, line no. 343.; 345.; 347-348, 356., Table 2.

**„add logical reasoning of each result”**

We revised the section „ Results and Discussion” and corrected.

Page no.22, line no. 434-438.

Page no.26-27, line no.557-560

**„should be brief and to the point”**

We revised „ Conclusion” and corrected.

Reviewer #2

**“Page no. 6, line no. 102: Separate “abovementioned”; it is jumbled.”**

Corrected.

**“Page no. 6, line no. 114: “Day old” means “first day of chick’s life”. Please avoid to mention “1-day old” from the entire manuscript.”**

Corrected in the entire manuscript

**“Page no. 7, line no. 134: Replace “1” with “single”. Replace “3 birds/pen” with “three birds/ pen”.**

Corrected.

**“Page no. 9, line no. 157: Replace “dH_2_O” with “distilled water” and make it uniform for the entire manuscript.”**

Corrected in the entire manuscript.

**“Page no. 13, line no. 256: Insert “.” before the word “Both”.**

Corrected.

**“Abstract section: “A method was developed to measure the levels of other small molecule thiol components of plasma”.**

**But in page 12, line 234: The free thiol concentration was determined through Ellman’s assay (Ethos Biosciences Inc., Philadelphia, PA, United States).Please justify the statement of abstract?”**

The free thiol concentration was determined through Ellman’s assay. We also determined the total thiol and disulfide content with a new method based on. concentration (protein bound and free) Xiaoyun Fu et al.’s publication

**“Page 13, line 260 – 264: If any new method cited, it should be described by the authors. In case already published method, reference could be cited and a brief account could be presented.”**

Corrected. Page no. 13-14, line no. 263-271

**“Avoid presentation of conclusion in bullet form. It should be presented in sentence case within word count of 150.”**

Corrected

PLOS ONE’s publication criteria

**Page no. 1, line no. 10. “*” inserted**

**Page no.5, line no.81. change citation „[22]”**

In references:

Page no. 34, line no. 750-751

22.	Wilson JB, Julian RJ, Barker IK. Lesions of Right Heart Failure and Ascites in Broiler Chickens. Avian Diseases. 1988;32: 246–261. doi:10.2307/159081

**Page no.6., line no. 120-121.**

We specified the material and method

**Page no.7., line no.130.**

We specified the material and method.

**Page no. 17, line no. 348, new citations [64,65]**

In references:

Page no.38, line no.880-884

64.	Al-Tamimi H, Mahmoud K, Al-Dawood A, Nusairat B, Khalaf HB. Thermotolerance of Broiler Chicks Ingesting Dietary Betaine and/or Creatine. Animals. 2019;9: 742. doi:10.3390/ani9100742

65.	Ndlela SZ, Moyo M, Mkwanazi MV, Chimonyo M. Responses of gut morphology, serum biochemistry, and quality of breast meat to water deprivation in broilers. Can J Anim Sci. 2020;100: 59–68. doi:10.1139/cjas-2018-0221

**Page no. 21, line no. 418. corrected citations [85,86]**

In references:

Page no. 39., line no. 941-947.

85.	Weaver K, Skouta R. The Selenoprotein Glutathione Peroxidase 4: From Molecular Mechanisms to Novel Therapeutic Opportunities. Biomedicines. 2022;10: 891. doi:10.3390/biomedicines10040891

86.	Giustarini D, Tsikas D, Colombo G, Milzani A, Dalle-Donne I, Fanti P, et al. Pitfalls in the analysis of the physiological antioxidant glutathione (GSH) and its disulfide (GSSG) in biological samples: An elephant in the room. J Chromatogr B Analyt Technol Biomed Life Sci. 2016;1019: 21–28. doi:10.1016/j.jchromb.2016.02.015

**Page no.22, line no.436.; 438., new citations [89,90]; [91,92]**

In references:

Page no. 40, line no. 956-963

89.	Assady M, Farahnak A, Golestani A, Esharghian M. Superoxide Dismutase (SOD) Enzyme Activity Assay in Fasciola spp. Parasites and Liver Tissue Extract. Iran J Parasitol. 2011;6: 17–22.

90.	Escobar JA, Rubio MA, Lissi EA. SOD and catalase inactivation by singlet oxygen and peroxyl radicals. Free Radical Biology and Medicine. 1996;20: 285–290. doi:10.1016/0891-5849(95)02037-3

91.	Baba SP, Bhatnagar A. ROLE OF THIOLS IN OXIDATIVE STRESS. Curr Opin Toxicol. 2018;7: 133–139. doi:10.1016/j.cotox.2018.03.005

92.	Pisoschi AM, Pop A. The role of antioxidants in the chemistry of oxidative stress: A review. European Journal of Medicinal Chemistry. 2015;97: 55–74. doi:10.1016/j.ejmech.2015.04.040

**Page no. 22, line no. 457.**

A reference cited multiple times with a different reference number, corrected and the repeated reference was deleted from the reference list

**Page no. 23,**

line no.466., There was an incorrect image citation, corrected

line no. 476-477., There were incorrect image citations, corrected

line no. 483., A reference cited multiple times with a different reference number, corrected and the repeated reference was deleted from the reference list.

**Page no. 25., line no.528.; 530.**

A reference cited multiple times with a different reference number, corrected and the repeated reference was deleted from the reference list.

**Page no. 27. line no. 560.; new citation, [135]**

In references:

Page no. 43., line no.1074-1076

135.	Taherkhani S, Suzuki K, Castell L. A Short Overview of Changes in Inflammatory Cytokines and Oxidative Stress in Response to Physical Activity and Antioxidant Supplementation. Antioxidants (Basel). 2020;9: 886. doi:10.3390/antiox9090886

**Page no. 29., line no. 613.,**

A reference cited multiple times with a different reference number, corrected and the repeated reference was deleted from the reference list.

**Page no. 30.,**

**line no.643-644.,** Missing figure citation, corrected „Fig. 6”

**line no. 657.,** A reference cited multiple times with a different reference number, corrected and the repeated reference was deleted from the reference list.

**Page no. 31. line no. 664**

A reference cited multiple times with a different reference number, corrected and the repeated reference was deleted from the reference list.

**Page no. 33. line no.692-693**

# We wrote a statement what role was the funders in Acknowledgments.

# “The funders had no role in study design, data collection and analysis, decision to publish, or preparation of the manuscript.”
